# Preventing opioid prescribing for low back pain using multimodal mechanical stimulation vs. TENS: a randomized-controlled trial

**DOI:** 10.3389/fpain.2025.1612572

**Published:** 2025-07-10

**Authors:** Amy L. Baxter, Jena L. Etnoyer-Slaski, Jessica Allia Rice Williams, Kevin Swartout, Lindsey L. Cohen, M. Louise Lawson

**Affiliations:** ^1^ Department of Emergency Medicine, Augusta University, Augusta GA, United States; ^2^ Harmonic Scientific LLC, Lewes, DE, United States; ^3^ Kaizo Clinical Research Institute, Landover, MD, United States; ^4^ Department of Health Policy and Administration, Penn State College, University Park, PA, United States; ^5^ Department of Psychology, Georgia State University, Atlanta, GA, United States; ^6^ Department of Statistics and Analytical Sciences, Kennesaw State University, Kennesaw, GA, United States

**Keywords:** opioid, M-Stim, DuoTherm, TENS, low back pain, multifidus, focal vibration

## Abstract

**Background:**

Low back pain (LBP) is the most common reason for outpatient opioid prescribing: a quarter of patients receive prescriptions, leading to opioid use disorder (OUD) in 5%. Guideline-recommended multimodal interventions often face implementation barriers, and effective modalities (e.g., electrical stimulation) lack coverage. A multimodal mechanical stimulation (M-Stim) device for LBP has demonstrated safety and efficacy in pain reduction, but its impact on opioid use has not yet been determined.

**Methods:**

As part of an NIH-funded double-blind study to reduce pain and opioid use, patients with moderate-to-severe LBP presenting to two suburban chiropractic centers were randomized to receive either the M-Stim device or a transcutaneous electrical nerve stimulation (TENS) unit for 30 min daily, in addition to other therapies. Analgesic use was reported daily for 28 days, with new prescribing followed weekly for 3 months. The primary outcome was prescribing in the opioid-naïve subjects. Secondary endpoints included risk factors for prolonged use in the opioid-naïve subjects, milligram morphine equivalents (MME) for opioid users between the first and last 2 weeks, and prescribing compared with national rates.

**Results:**

After informed consent, 159 eligible patients were randomized to M-Stim (87) or TENS (72) (mean age 42.6 years, 54% female, BMI 30.9, NRS 5.5) between 23 June 2022 and 31 December 2023. Zero opioid-naïve M-Stim participants (*n* = 43) received prescriptions (0% vs. 8.6%, Fisher's exact *p* = 0.086), and those taking opioids used significantly fewer MME [7.5 (SD 3.54) vs. 498.5 MME (SD 474.9), *p* < 0.0001] for fewer of reported days [M-Stim 2/47 (4.2%)] compared with TENS [*n* = 36, 38/102 (37%), RR 0.11 (95% CI 0.28–0.44), *p* = 0.0018]. M-Stim significantly reduced MME in opioid users [−44.6% (32.33 MME), *p* = 0.02], use days for those with BMI ≥30 [−3 (99% CI −5.73 to −0.26), *p* = 0.032], and prescribing compared with national rates [9.8% vs. 25%, −63%, RR 0.32 (95% CI 0.16–0.66), *p* = 0.002] while TENS did not.

**Conclusions:**

Among chiropractic patients with moderate-to-severe LBP, added use of a multimodal M-Stim device in the opioid-naïve subjects significantly reduced factors associated with OUD compared with TENS and reduced use days for those with BMI ≥30. This novel device is a potential alternative to prescribing opioids as first line for LBP management.

**Clinical Trial Registration:**

https://clinicaltrials.gov/study/NCT04491175, identifier NCT04491175.

## Introduction

Low back pain (LBP) affects 80% of Americans during their lifetimes (1), 60% each year, and is estimated to impact 57 million people in North America by 2050. Although opioid prescribing has declined in response to the national emergency, LBP remains the most common reason for ambulatory opioid prescribing, with 25%–32.5% of moderate-to-severe pain patients receiving prescriptions (2–4). As with other acute pain conditions, 5% of those prescribed opioids develop prolonged use (5, 6). In an opioid-naïve population, dose, duration, and use beyond 7 days are risk factors for progression to opioid use disorder (OUD) (6, 7). Among chronic opioid users, fear of pain is the primary barrier to reducing use (8). To prevent OUD and improve LBP outcomes, better pain relief alternatives are critically needed (9–11).

While clinical guidelines recommend non-pharmacologic approaches first (12, 13), delivery of effective alternatives at point of care is problematic. Physical therapy (14), spinal manipulation (15), fascial treatments, or acupuncture require both coverage and rapid referral (3). Approaches combining multiple patient-controlled modalities are most effective (16), but non-drug interventions are not part of standard medical curricula, and explaining multimodal options to patients is time-consuming. Moreover, evidence-based non-pharmacologic LBP modalities, such as thermal treatments, yoga, transcutaneous electrical stimulation (TENS) (17, 18), and high-frequency vibratory mechanical stimulation (M-Stim) (19–24), are excluded from ambulatory coverage: Medicare explicitly denies payment for “personal comfort” pain relief interventions [section 1862(a) of the Social Security Act] (6). Thus, time, familiarity, and cost considerations contribute to physicians prescribing opioids over multimodal options (13, 16, 25).

To bridge this gap, the National Institute on Drug Abuse (NIDA) funded the development of a novel non-invasive multimodal heat, pressure, and harmonic multifrequency vibration device as an opioid alternative. In a Phase 1 pilot study (26), this M-Stim device reduced LBP by 57% after 20 min. The present double-blind active-controlled trial tested the hypothesis that moderate-to-severe LBP patients would be less likely to seek out and use opioids with a multimodal pain relief device than an established single-modality active control. The primary outcome was reduced prescribing in the opioid-naïve subjects compared with TENS over 3 months. Secondary endpoints included the reduction of risk factors associated with prolonged use in opioid-naïve subjects, including milligram morphine equivalents (MME) and days of use. Because of the potential for reduced prescribing due to dispensing any device, additional outcomes included prescribing compared with historical national rates and change in MME for opioid users tracked daily between the first and last 2 weeks.

## Methods

### Device description

**Figure 1:**
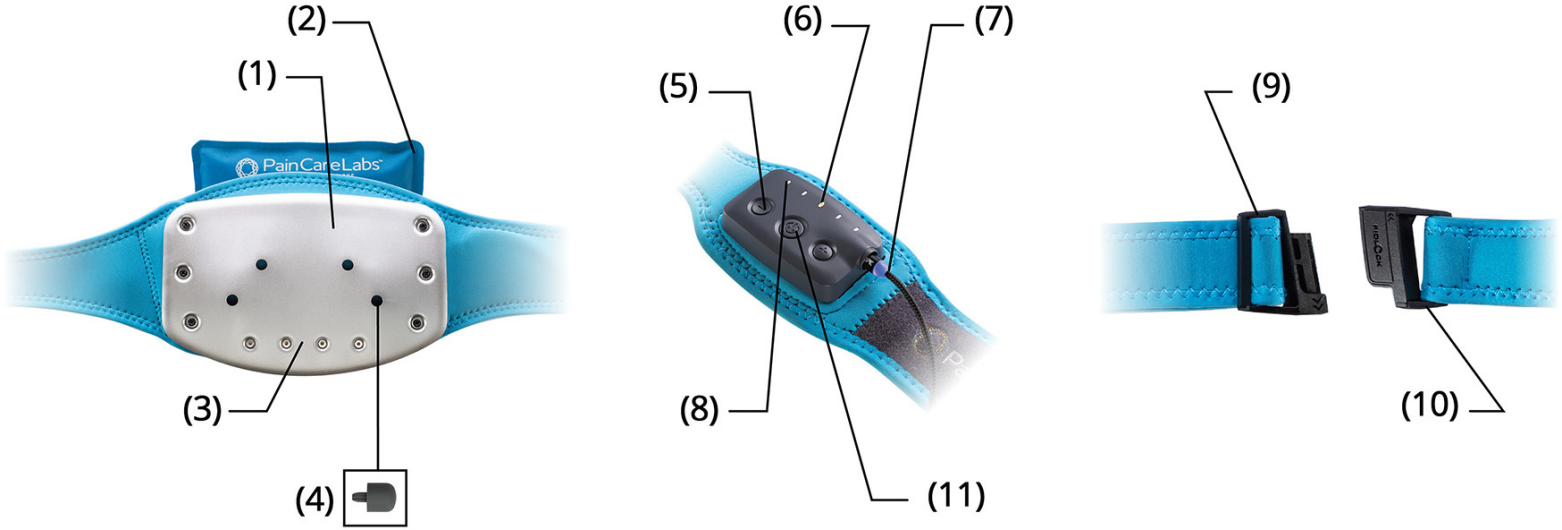
Contoured temperature plate (1). Natural clay ice/heat pack (2). Multi-vibration motor array (3). Trigger point acupressure nubs (4). Five intensity settings (5). LED cycle and intensity display (6). Magnetic charging cable (7). Eight therapy cycles (8). Custom-fit waistband (9). Slide-N-Lock magnetic buckle (10). Haptic touch control panel (11).

Non-specific mechanical low back pain has no definitive radiologic findings (27) and is variably attributed to dysfunction of the paraspinal muscles, neuromotor instability, and fascial inflammation (28), with central pain sensitization and psychologic components (29, 30). The M-Stim device delivers mechanical force to the thoracolumbar field through a 6″ × 8″ thermoconductive metal plate held by a 54″ compressive neoprene belt (26). The plate was first described by Lundeberg (31), who reported that a single 100 Hz or 200 Hz motor on a 6″ × 8″ flat plate reduced low back pain more effectively than TENS. The lower frequency has since been shown to improve neuromotor reflexes and proprioception (32), while the 200 Hz Lundeberg found more effective activates spinal gating to reduce sharp pain transmission (33). To concentrate the vibratory mechanical force, curves on the M-Stim device amplify pressure on the paraspinal muscles. Three motors in harmonic frequencies (50 Hz, 100 Hz, and 200 Hz) with amplitude 0.03 m/s^2^–0.1 m/s^2^ interact in eight possible therapeutic cycles programmed to deliver stochastic force in frequencies associated with myriad tissue effects [e.g., oxytocin release (34), reduction of fatty changes (35), decreased inflammation and disc degeneration (36), and vasodilation (37)]. To activate force-gated Piezo1 and Piezo2 ion channels mediating these processes at variable tissue depths (38–41), constructive interference between frequencies enhances the amplitude for deeper mechanical energy penetration. Five intensity settings increase both amplitude and frequency (Figure 1).

Fascial tissue gliding is enhanced with heat (42), while cryotherapy decreases inflammatory cytokine production (43). Thus, thermal heat and cold packs can be placed behind the plate. Pressure enhances fascial glide and forces extracellular liquid into gliding hyaluronic complexes, so the holes in the plate allow for four different locations of a 1.5 cm silicone acupressure nub.

To reinforce the device's benefit as a part of reducing central sensitization (25), the 200 Hz frequency reduces nociceptor firing on contact (44–46), while heat can be comforting or reduce spasm. The device is easy to apply to reduce catastrophizing and helplessness, while the multiple settings and options are intended to improve self-efficacy (feeling empowered to choose), all of which are associated with reduced opioid intake (47).

### Study design

This randomized double-blind active-controlled trial was designed to evaluate the efficacy of multimodal M-Stim compared with TENS to reduce opioid prescribing and pain in patients with moderate-to-severe LBP [≥4 on a 0–10 numeric rating scale (NRS)]. Between June 2022 and December 2023, 160 adults aged between 20 and 75 years were recruited at two chiropractic and motor vehicle collision (MVC) physical therapy referral clinics in Maryland and Virginia, with follow-up completed in July 2024. The sites had no access to practitioners who could prescribe opioids. Enrollment was stratified by chronicity for pain outcomes [chronic (cLBP) ≥3 months (*n* = 100) or acute (aLBP) <3 months (*n* = 60)] with opioid use tracked daily for 28 days and then weekly for 3 months. The exclusion criteria included radicular pain, sickle cell disease, sensitivity to cold or vibration, a pacemaker, skin lesions in the low back, or inability to apply the devices as directed (Supplementary Material S1). The Kaizo Clinical Research Institutional Review Board approved the trials, which were registered with ClinicalTrials.gov NCT04491175 and monitored for severe adverse events.

### Interventions

Participants were randomized to add 30 min daily use of a prescription eight-channel electrical stimulation TENS unit (LG Smart TENS, LGMedSupply, Cherry Hill, NJ, USA; $125) or multimodal M-Stim (DuoTherm, Harmonic Scientific LLC, Lewes, DE, USA; reimbursement not yet determined) to any other treatments. A text and email prompted participants to record analgesic use, pain, and device use daily for 28 days, with prescribing outcomes followed weekly for 3 months.

The TENS unit delivers electrical stimulation via four electrodes with eight use channels, adjustable intensity, and duration. A systematic review reported TENS' immediate reduction of NRS pain intensity for both acute and chronic LBP at a standardized mean difference (SMD) of −0·96 (95% CI −1.14 to −0.78) (17), while a review of vibration for cLBP found reduced pain intensity SMD = −0.71 (95% CI −1.02 to −0.39) (23).

### Procedure, randomization, and assessment

Prior to treatment, clinic intake staff assessed eligibility and completed informed consent for the study “to evaluate the effect of an electric or mechanical stimulation device on opioid use and pain relief.” All patients provided written informed consent.

After consent, a Qualtrics link on the data intake tablet randomly generated a study ID and coded device assignment with no blocking or additional stratification. Baseline pain intensity was recorded prior to learning the device assignment. After participants received their device, they watched the appropriate training video (Supplementary Material S1) for DuoTherm or LG-TENS and then initiated a 30 min use while completing registration entry. Registration data include the NIH Minimum Data set for low back pain studies (48), including the PROMIS Pain Intensity (4a), Physical Function (4a), Pain Interference (8a), Depression, Sullivan Pain Catastrophizing scale, and an inventory of 13 prior treatment options including cannabis and gabapentin.

Prior opioid use at registration was assessed with the questions “What short-acting/long-acting medications have you taken for your pain in the past? (Choose all that apply)” with a comprehensive list of opioid formulations and doses (DOSE tool, Supplementary Material S2). Daily and weekly opioid diary prompts used the short-/long-acting formulation questions, adding dose per pill (34), number of pills, and source. Sources presented in order were “prescribed to me for this event,” “prescribed to me for another event,” “given to me by a family member,” “given to me by a friend,” “given to me by an acquaintance or stranger,” or “purchased from someone without a prescription.” Clinic staff documented treatments received after enrollment.

### Outcomes

The primary outcome was reduction in opioid prescribing to the opioid-naïve subjects. Opioid use status was considered “naïve” with no endorsement of any listed opioids and endorsement of “I have not taken any of these short-acting/long-acting medications in the past” at registration; otherwise participants were considered “prior users.” Receiving a prescription was a binary self-reported outcome where any opioid recorded as “prescribed to me for this event” was considered a new prescription. Secondary outcomes associated with a higher risk of subsequent OUD included self-reporting of milligrams of morphine equivalents, days of use, use in the past 7 days, and receiving a prescription 7 days after presentation (6). Quantitative MME were calculated from the reported opioid DOSE diaries using the health and human services conversion tables (3, 4, 49, 50).

To explore opioid use for chronic users, we established a baseline of use for the first 14 days and compared it to use during the second 14 days using MME as an indicator of pain reduction. Additional outcomes included prescribing rates compared with a national rate of 25%. ALBP pain relief and cLBP disability study outcomes are presented elsewhere.

### Blinding assessment

Participants knew the study was intended to evaluate opioid use, but were blinded to whether the electrical or mechanical stimulation device powered the study hypothesis. The protocol statistician (KS) and study coordinator (JE-S) knew device assignments and had access to data, but did not conduct analysis. The PI (AB) and treating chiropractor (AH) made no outcome judgments and were blinded to allocation and all data during enrollment, with the PI accessing data only after study completion and data lock. The analyzing statisticians (JW, OT) were blinded to device assignment until the completion of analysis. The success of participant blinding was tested at 3 months with prompts, “What was the study trying to find out?”, “Select if you received…control or treatment”, and “How confident are you?”

### Sample size calculation

At the time of study conception, no non-invasive opioid-reducing device research was available to estimate the effect size for a power analysis to prevent opioid prescribing. A retrospective spinal stimulation study of 59 cLBP chronic opioid users reported a 28% MME reduction after implantation (effect size 0.60) against standard care (51). Using this effect size for a two-sided significance of 0.05, 23 participants per group would detect a 30% prescribing reduction for our primary opioid outcome, similar to the 28% MME reduction. We planned to recruit 60 opioid-naïve subjects for prescribing outcomes anticipating a 10% attrition. Our primary pain outcome of treating disability powered the cLBP recruitment of 100 patients. However, if 60% of cLBP patients had chronic or ongoing opioid use (7), it would replicate the number of opioid users (59) in the spinal stimulation study.

### Analysis

**Figure 2:**
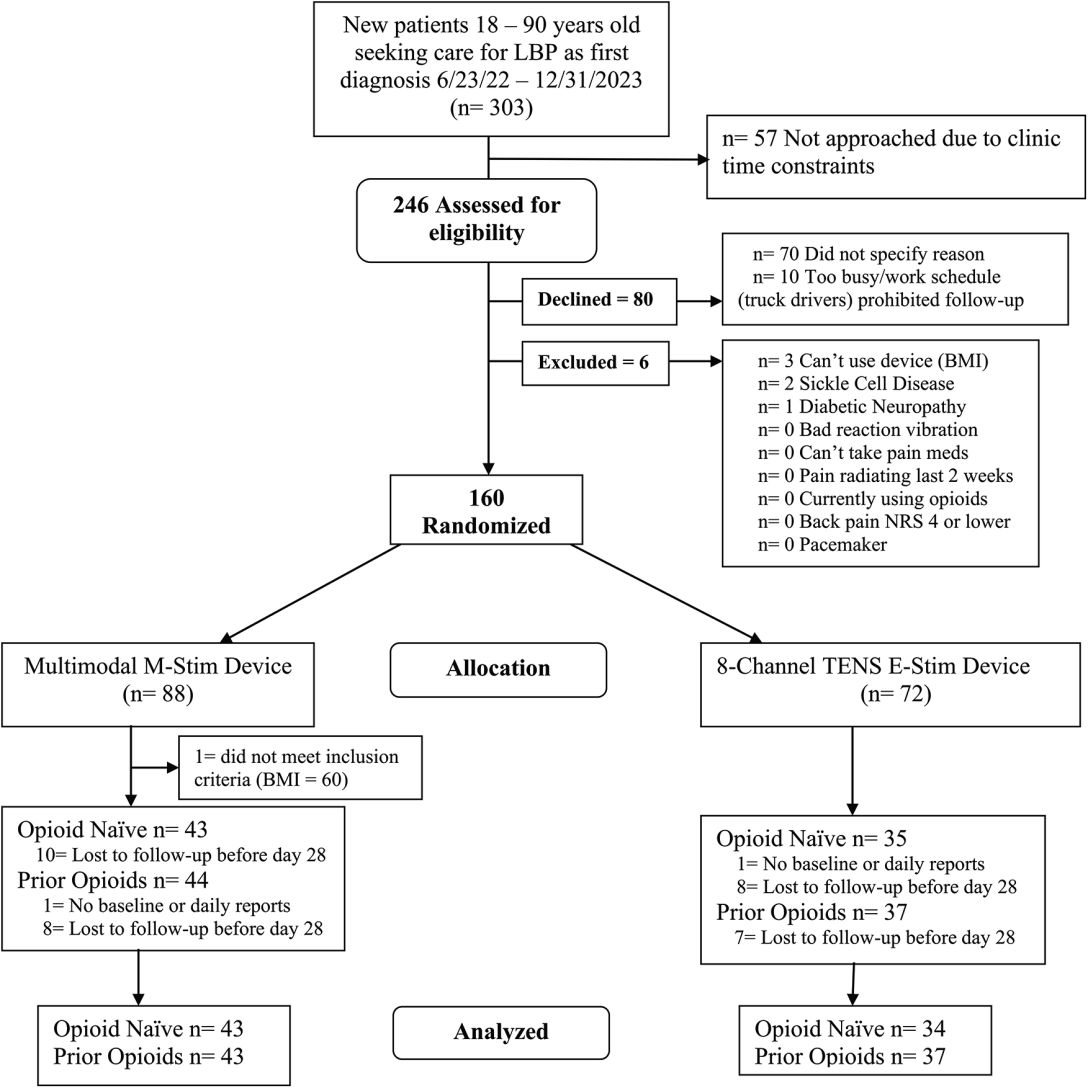
The consort flowchart opioid use low back pain.

Opioid prescribing differences were calculated with percentages and relative risks for binary outcomes, using Fisher’s exact for cells with zero. Opioid use days included summary statistics (means, standard deviations) and Chi-squared tests. Paired *t*-tests were used to compare the change between the first and the last 14 days of MME for opioid users. As the number and missing data patterns of the diary entries and opioid use days did not statistically differ between intervention groups, we did not impute missing opioid use (missing data Supplementary Material S4) but instead calculated a linear mixed model (LMM) for the first month with daily reporting for all participants with at least one opiod diary input as a baseline (Figure 2). For demographic and pain history factors that differed between groups, we planned a regression analysis to assess any interaction of characteristics with opioid use. The two-tailed significance level was set at 0.05%, and 95% confidence intervals were reported. Data analysis was performed using StataNow/SE 18.5 and MedCalc Software Ltd. (https://www.medcalc.org/calc/fisher.php; Version 23.2.1; accessed 3 April 2025).

## Results

### Participants

We enrolled 160 participants, of whom 159 were eligible (M-Stim = 87, TENS = 72) (Figure 2). One enrolled M-Stim patient (BMI = 60) was unable to apply the device. The majority were female (54.7%), non-Hispanic (94.3%), and Black or Multiple race (72.9%). The average age was 41.1 years (SD = 12.2), BMI 30.9%, and 31% had a household income of <$75,000. The average initial pain intensity was 5.46 on the 0–10 NRS, with a PROMIS Pain Interference T-score of 63.65 (high moderate severity). Based on registration reporting, 78 (49%) denied prior or current opioid use for back pain (“opioid-naïve”), while 81 (51%) acknowledged prior or current use (“prior user”). Demographic and pain history did not differ significantly by intervention group for 51 of the 54 data categories. The TENS group had a higher average BMI [32.04 (7.05) vs. 29.88 (5.22), *p* = 0.028], whereas the M-Stim group reported baseline PROMIS “worst pain intensity during the past 7 days” at 0.26 higher on a 1–5 scale and were less likely to endorse using vibration for prior pain management (Table 1). One participant per group filled no diary entries; 68 (78.2%) M-Stim and 60 (77.8%) TENS users reported through at least 1 month. Diary entry frequency did not differ by intervention, duration, or opioid use status. There were no reported device-related or significant adverse events.

**Table 1:** Sociodemographic and clinical characteristics.

Characteristic	Total (*n* = 159)	DuoTherm (*n* = 87)	TENS (*n* = 72)
Age in years, mean [SD]	42.58 [12.31]	40.85 [11.97]	44.67 [12.47]
Female [No. (%)]	86 (54.09)	48 (55.17)	38 (52.78)
Race			
Black	106 (66.67)	60 (68.97)	46 (63.89)
White	23 (14.47)	10 (11.49)	13 (18.06)
AI/AN, Asian, or Hawaiian/PI	10 (6.29)	5 (5.75)	5 (6.94)
Unknown/Not Reported	9 (5.66)	6 (6.90)	3 (4.17)
Multiple	11 (6.92)	6 (6.90)	5 (6.94)
Ethnicity—Hispanic or Latino	12 (7.55)	3 (3.45)	9 (12.5)
Marital status			
Married/domestic partner	79 (49.69)	38 (43.68)	41 (56.94)
Never married	62 (38.99)	37 (42.53)	25 (34.72)
Divorced/separated/Wwidowed	18 (11.32)	12 (13.79)	6 (8.33)
Highest complete education			
Less than high school	10 (6.29)	6 (6.90)	4 (5.56)
High school	48 (30.19)	24 (27.59)	24 (33.33)
Associates or technical degree	20 (12.58)	9 (10.34)	11 (15.28)
Baccalaureate or higher	81 (50.94)	48 (55.18)	33 (45.84)
Household income			
Less than $75 K	38 (23.9)	19 (21.84)	19 (26.39)
At least $75 K	83 (52.2)	49 (56.32)	34 (47.22)
Prefer not to answer	38 (23.9)	19 (21.84)	19 (26.39)
Not employed	26 (16.35)	14 (16.09)	12 (16.67)
Average size of household	2.72 (1.42)	2.69 (1.51)	2.75 (1.31)
Substance use			
Current smoker (Y) (PCORI)	19 (11.95)	9 (10.34)	10 (13.89)
Drunk/used drugs more than you wanted (> “never”)	33 (20.75)	13 (14.94)	20 (27.78)
Wanted/needed to cut down on drinking/drug (> “rarely”)		6 (6.90)	7 (9.72)
Back pain history			
*Duration of low back pain*			
Acute (<3 months)	44 (27.67)	22 (25.29)	22 (30.56)
Chronic (≥3 months)	115 (72.33)	65 (74.71)	50 (69.44)
Chronic (>5 years)	62 (38.99)	34 (39.08)	28 (38.89)
History of MVC	61 (38.36)	33 (37.93)	28 (38.89)
Previous back operation (Y)	9 (5.66)	6 (6.90)	3 (4.17)
Spinal fusion (Y)	3 (1.89)	2 (2.30)	1 (1.39)
Back injections (Y)	29 (18.24)	18 (20.69)	11 (15.28)
Filed a workers' compensation claim for LBP? (Y)	12 (7.55)	8 (9.20)	4 (5.56)
Involved in legal claim related to LBP? (Y)	34 (21.38)	18 (20.69)	16 (22.22)
Filed for disability due to LBP? (Y)	13 (8.18)	10 (11.49)	3 (4.17)
*Pain prescriptions*			
Prior opioid use for back pain (Y)	80 (50.31)	44 (50.57)	36 (50.00)
Prior or current gabapentin (Y)	17 (10.69)	9 (10.34)	8 (13.89)
TENS (Y)	47 (29.56)	24 (27.59)	23 (31.94)
Vibration^a^ (Y)	60 (37.73)	27 (31.03)	33 (45.83)
Cannabis (Y)	21 (13.21)	11 (12.64)	10 (13.89)
Cognitive behavioral therapy (Y)	10 (6.29)	4 (4.60)	6 (8.33)
Exercise at least once weekly	124 (76.19)	67 (75.61)	57 (76.92)
Tried ≥5 of 11 integrative treatments	53 (33.33)	26 (29.89)	27 (37.5)
Physical			
BMI^a^, mean [SD]	30.86 [6.19]	29.88 [5.22]	**32.04 [7.05]**
BMI by category (%)			
<25	26 (16.35)	14 (16.09)	12 (16.67)
25–29.9	55 (34.59)	34 (39.08)	21 (29.17)
≥30	78 (49.06)	39 (44.83)	39 (54.17)
Current back pain characteristics			
Pain at least half of the days (Y)	127 (79.87)	70 (80.46)	57 (79.17)
Pain intensity now NRS_1^b^ 0–10 [SD]	5.51 [2.15]	5.53 [2.02]	5.48 [2.32]
Pain intensity 24 h NRS_2^c^ [SD]	6.39 [2.19]	6.5 [1.99]	6.25 [2.42]
PROMIS Pain Intensity^a^ (7 days worst 1–5) [SD]	4.02 [0.79]	**4.14 [0.73]**	3.88 [0.84]
PROMIS 7 days average (1–5) [SD]	3.22 [0.89]	3.22 [0.75]	3.22 [0.89]
PROMIS Pain Interference T-Score (normed at 50, higher worse) [SD]	63.65 [7.18]	63.56 [5.91]	63.75 [7.18]
PROMIS Physical Function T-Score (normed at 50, lower is worse) [SD]	36.57 [0.40]	36.32 [4.67]	36.87 [5.55]
PROMIS Depression T-Score (normed at 50, higher is worse) [SD]	50.40 [0.77]	50.28 [1.03]	50.55 [1.18]
Catastrophizing > 22 (out of 52)	21.45 (13.71)	21.51 (13.87)	21.38 (13.63)

^a^ *P* < 0.05, higher risk factors associated with LBP in bold.

^b^ Missing one score in the TENS group.

^c^ Missing one score per group.

### Opioid prescribing and use in the opioid-naïve

In the opioid-naïve cohort, zero M-Stim users (*n* = 43) received prescriptions compared with 3 of 36 using TENS (0% vs. 8.6%, Fisher's exact *p* = 0.086) (Table 2). For those who did take opioids, the M-Stim group averaged significantly reduced MME [7.5 (SD 3.54) vs. 498.5 MME (SD 474.9), *p* < 0.0001] with a lower risk of taking opioids on any daily entry: 2/47 (4.2%) using M-Stim compared with 38/102 (37%) entries by TENS users [RR 0.11 (95% CI 0.28–0.44, *p* = 0.0018, NNT 2.9]. All participants who took opioids in the TENS group used opioids after 7 days (*p* = 0.086); two TENS users (5.5%) had the additional OUD risk factor of opioid use for 7 or more days (*p* = 0.20). The two M-Stim participants each took one opioid on 1 day prior to day 7, obtained from sources other than a new prescription (Table 2).

**Table 2:** Opioid prescribing and MME among subjects with diary entries by use and pain status.

Panel A. Opioid prescribing and MME use among enrolled opioid-naïve subjects (*n* = 78)			
Metric	M-Stim (*n* = 43)	TENS (*n* = 35)	*p*-value
New prescription [No. (%)]	0 (0%)	3 (8.6%)	*p* = 0.086
Any opioid use	2 (4.7%)	3 (14.3%)	NS
Average MME if any opioid use	7.5 (SD 3.54)	498.5 (SD 475)	Welch’s *t*-test for the difference (df = 33): ***p* < 0.0001**^c^**, *t*-statistic = −6.03**
Use the past 7 days	0 (0%)	3 (8.6%)	*p* = 0.086
Use for 7+ days	0 (0%)	2 (5.7%)	*p* = 0.20
Diary entries (total = 2,210)	1,217 (Avg. 28.3)	993 (Avg. 28.4)	NS
Entries by opioid users	47 (Avg. 23.5)	102 (Avg. 34)	NS
Days with opioid use	2 (4.7%)	39 (38.2%)	**RR = 0.11, 95% CI (0.028–0.44), *p* = 0.0018, NNT 2.9** ^c^
Panel B. Opioid prescribing and MME use among opioid users during study (*n* = 32)			
Metric: average	M-Stim (*n* = 14)	TENS (*n* = 18)	**Difference**
Average MME use for the first 14 days of the month	88.65 MME	46.22 MME	**56.9% reduction** ^b^
Average MME use for the last 14 days of the month	56.32 MME	52.26 MME	
Paired *t*-test for difference between the first and last 14 days	**−32.33 *p* = 0.02**	+6.04 *p* = 0.79^a^	
	**(44.6% less)** ^c^	(12.3% more)	
Diary entries (total = 937)	398 (Avg. 28.4)	539 (Avg. 29.9)	NS
Panel C. Opioid prescribing and MME use among all subjects with diary entries (*n* = 157)			
Metric: number (%)	M-Stim (*n* = 86)	TENS (*n* = 71)	Relative risks and significance
New prescription	8 (9.3%)	11 (15.5%)	**41.7% reduction** ^b^
			RR = 0.60, 95% CI (0.26–1.41), *p* = 0.24, NNT 16.2
Any use	14 (16.3%)	18 (25.4%)	RR = 0.64, 95% CI (0.34–1.20), *p* = 0.16, NNT 11.0
Prescribing compared with national 25%	**−63%, RR 0.32, 95% CI (0.16–0.66), *p* = 0.002, NNT 5.97** ^c^	−38%, RR 0.62, 95% CI (0.36–1.09), *p* = 0.098, NNT 10.86	

Statistically significant results in bold.

^a^ For the change in use over the duration of daily diary collection, one outlier in the TENS group increased MME in the second half of reporting but was retained in the statistical calculations.

^b^ Achieved primary outcome milestone.

^c^ Statistically significant.

### Opioid prescribing and use overall

**Figure 3:**
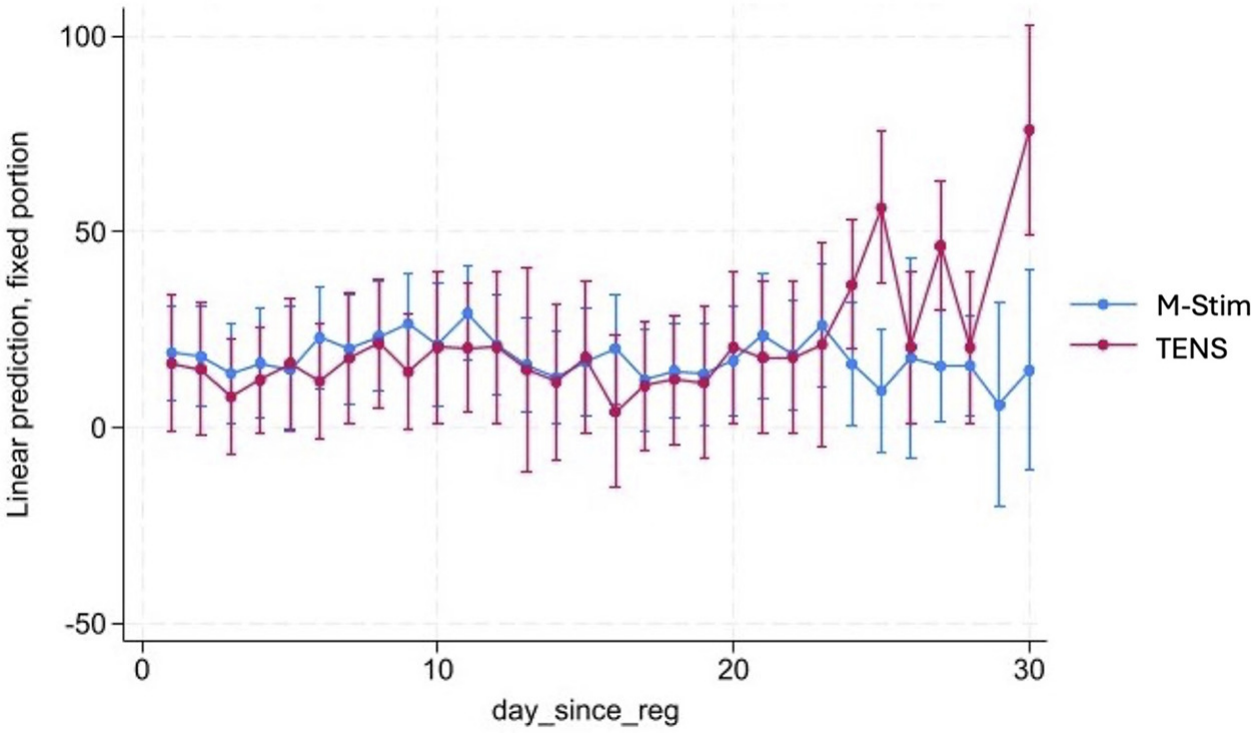
Linear mixed-effects model of MME change by opioid users. To evaluate opioid use patterns in the first month of intervention, we conducted a linear mixed-effects model (LMM-REML) examining daily milligram morphine equivalents (MME) for anyone with at least one positive MME value over 28 days, including the 30-day month diary timepoint reporting for the previous two days. Fixed effects included time (day since registration), condition [M-Stim (Condition 1) vs. TENS (Condition 2)], and their interaction (condition x time). A random intercept was induced for each participant to account for within-subject variability. The RE parameters were statistically significant, 106.58, 95%CI [61.56, 184.52]—suggesting substantial individual variation. There was also substantial variation that was not explained by the model. An LR test was statistically significant (*P* < 0.001)—indicating better fit than a single-level model. The effect of condition 1 versus condition 2 was not statistically significant.

M-Stim users were 41.7% less likely to receive new prescriptions compared with TENS [7 of 86 (8.14%) vs. 11 of 71 (15.4%), RR 0.51, 95% CI 0.2099–1.256, *p* = 0.1441], which was not statistically significant (Table 2). Compared to national 25% rates, dispensing an M-Stim device significantly reduced prescribing (RR 0.3220, 95% CI 0.1570–0.6604, *p* = 0.002, NNT 5.971), while TENS devices did not (RR 0.6272, 95% CI 0.3608–1.094, *p* = 0.0982, NNT 10.858) (Table 2). Among 32 opioid users, M-Stim significantly reduced opioid use by 44.6% (32.33 MME, *p* = 0.02) while TENS users experienced a 12.3% increase (+6.04 MME, *p* = 0.79), but this was primarily due to one outlier (Figure 3).

### Sources of opioid use

Of 4,124 diary entries, 801 opioid pills were reported among 263 diary entries, with 766 (95.6%) listing a source. In each intervention group, seven participants used opioids from sources other than new prescriptions (Table 3). Moreover, 28.8% of opioids reported were from prior prescribing rather than new prescriptions.

**Table 3:** Source of opioids from opioid diary entries.

Subjects reporting opioid use: 32 (20.38%)		Entries reporting opioid use: 263 of 4,124 entries
Opioid sources^a^		Source^b^ of 801 pills consumed
New prescription for this event	19	458
Used prescription from another event	12	231
Bought without prescription	2	66
Friend	2	8
Family	2	3
Brand/dose/pill #, no source reported	5	35 pills taken on 11 diary entries^c^
	Reported opioid source: 252/263 diary entries (95.82%)	

^a^ Eight subjects reported multiple sources for opioids.

^b^ Some reported multiple opioids on the same day, some with different sources, e.g., a previously prescribed and newly prescribed opioid on the same day.

^c^ These five participants did not report a source for any medication.

### Blinding assessment and impact of BMI

The most common initial response in both groups was to answer the study purpose question without endorsing an allocation, which we considered “I don’t know.” Blinding guesses were made by 56 in the M-Stim group (8, control; 29, don't know; 19, treatment) and 49 using TENS (12, control; 26, don't know; 11, treatment). Using Bang's blinding index, M-Stim BBI = −0.032 (95% CI −0.54 to −0.06), and TENS BBI = −0.51 (95% CI −0.71 to −0.24), both significantly against their allocation groups. Using the James index, M-Stim = 0.54, and TENS = 0.71, where values >0.5 indicate adequate blinding.

Because BMI was significantly higher in the TENS group, opioid use and days of use were evaluated including BMI in a logistic regression model. While adding BMI did not significantly change results for MME, the opioid-naïve subjects with a BMI ≥30 had 3.0 fewer days of opioid use in the M-Stim group compared with the TENS group (99% CI −5.73 to −0.26, *p* = 0.032). For those with BMI ≥30, the percent of days using opioids vs. all days was 9.65% lower in the M-Stim group compared with the TENS group (99% CI −19.5% to −0.79%, *p* = 0.033).

## Discussion

In the opioid-naïve subjects, the use of an M-Stim device significantly reduced risk factors associated with OUD for those who initiated opioids, including MME and days of use. Overall, opioid users reduced MME over the first 28 days significantly more using M-Stim than TENS, and M-Stim significantly reduced opioid use days for those with BMI ≥30. M-Stim users were prescribed opioids (8.14%) significantly less than the national moderate-to-severe LBP prescribing rate of 25%, but the 41.7% reduction compared with TENS was not statistically significant.

Low back pain is the leading cause of disability worldwide (52) and contributes $200–$600 billion in annual US healthcare costs (53–56). Risk factors for aLBP transitioning to cLBP include female sex (3), pain interfering with daily activities (48), low self-efficacy (helplessness), fear of pain (catastrophizing) (57), motor vehicle collision etiology (10), and receiving an opioid prescription (9, 58, 59). While LBP opioid prescribing fell after the declaration of the national crisis, LBP remains the most common reason for outpatient opioid prescribing in the USA (2, 60).

While current guidelines call for multimodal interventions instead of opioids (61, 62), alternative coverage and opioid familiarity inhibit physician recommendations (18, 63, 64). Immediate referrals work in other countries, but in the USA, the Patient-Centered Outcomes Research Institute (PCORI) declared that “implementation in practice has failed.” PCORI undertook a prospective trial in 76 clinics to reduce the 40% progression of aLBP to cLBP (3). After stratifying risk and providing services at the time of acute care presentation, this too failed, with 40% of moderate-to-severe aLBP still progressing to cLBP. Opioid prescribing was unchanged at 25%. Rather than referrals, effective devices are logical opioid-sparing interventions to dispense at presentation, balancing the physician's desire to act with the patient's impatience for relief.

The concept behind the DuoTherm device was initially to address pain with an evidence-based all-in-one neuromodulatory and thermal/pressure construct for easy prescribing at point of care. The device incorporated opioid reduction literature by reducing fear and increasing self-efficacy—factors associated with reduced chronic pain and OUD (25, 65, 66)—through multiple options and ease of use. Combining effective therapies of heat, cold, and pressure has synergistic effects on fascia, a common source of LBP (42). More recent discoveries, however, support the interplay of stochastic vibrations for vasodilation and mechanical force ion channel activation to reduce and address the muscle dysfunction associated with cLBP (67). The 200 Hz neuromodulatory frequency acutely blocks nociception via adenosine presynaptic inhibition in the dorsal horn, but is likely an additive rather than primary effect (33, 68). The range of frequencies applied across the entire thoracolumbar area address physiologic derangements from hypoperfusion to fatty changes (23), potentially providing pain relief by reversing newly understood mechanical causes of cLBP (30, 34, 69, 70). As such, M-Stim’s reduction of prescribing and opioid use may reflect processes other than reducing nociception.

LBP and opioid use are reciprocally linked to increased costs and disability: LBP increases the risk of OUD after surgery (71), and half of those on chronic opioids have LBP (7). The nature of opioid metabolism is implicated in the development of chronic pain. Opioids act on mu-opioid receptors (MORs), leading to antinociception through dopamine rewards. The receptors bring morphine into the cell and then return to the surface (72). After 3 days of opioid use, the receptors are more likely to be digested instead of returning; also around that time, increasing protein kinase C inhibits pain relief from opioids that are internalized (73, 74). In the face of decreased nociception, plastic processes in the brain increase pain perception to compensate for opioid-induced reduced sensitivity to physical risk (73). TENS acts in part via naloxone-reversible endogenous opioids, while vibration does not (31). If the receptors lose potency for exogenous opioids, reduced endogenous TENS efficacy might be expected after 3 days as well.

While no M-Stim opioid-naïve subjects were prescribed opioids, the rate for TENS users was only 8.7%, and not statistically significant. This may be clinically significant, however. The lead author of an international multidisciplinary team convened to address growing LBP prevalence (48) reported that one in 20 opioid-naïve patients prescribed opioids developed OUD (5), as did Hayden et al. (6) This OUD risk reporting is agnostic of pain etiology, whether for iatrogenic adult pain (75) or adolescent wisdom tooth removal (6.4%) (76). The current theory of a “reward deficient” genetic predisposition (77, 78) suggests that while reducing chronic opioid use is important, preventing initial prescribing is the critical goal. While not followed past typical time frames for prolonged use, 5% of our opioid-naïve TENS group had prolonged duration, late prescribing, and high MME risk factors for ongoing OUD. Using 2018 data, the mean value of averting one case of opioid misuse was estimated at $2.2 million, $325,125, and $244,030 in US societal, taxpayer, and healthcare costs, respectively (79). With 65 million LBP episodes yearly in the USA (52), if 25% of the 16.25 million seeking LBP relief receive opioids, the cost of 5% (203,125) becoming new prolonged opioid users is substantial.

To assess opioid use for outpatient low back pain, studies use prescribing (25%) or filled prescriptions (24.4%) (3, 6) as the accepted proxy, with a recent study confirming that all of the 94.2% filling prescriptions used them (80). Our study suggests this metric may underreport opioid consumption. Indeed, only 458 pills of 801 reported were “prescribed to me for this event,” implying that 42.8% of opioids would have been missed by validating against pharmacy records or prescription databases. While friends or family sources typically resulted in few use days, the two subjects who “bought without a prescription,” likely from an extra-legal source, averaged 10 days. Unused opioids from previous events were the second most common source after new prescriptions. As 90% of US households report having unused opioids (81), the prescription-only analyses are likely to underreport.

While there was no sham or placebo arm, the overall trend toward the superiority of M-Stim compared with TENS may be more relevant in light of TENS' established efficacy for pain intensity. In contrast, studies show subjects assume vibration works due to distraction (82). Participants using TENS did not appear to perceive their assignment as inferior; the James index of 0.71 and a Bang index of −0.51 suggest effective blinding leaning toward a more positive expectancy bias for TENS than M-Stim. That said, our average subject was obese. M-Stim reduced opioid use days significantly more in obese subjects, but increased adiposity may have confounded TENS' pain relief.

Future research should study TENS for opioid reduction against a sham or standard care, as it too may reduce unnecessary prescribing at a lower cost. As TENS and M-Stim work via different mechanisms, future devices could combine endogenous opioid TENS relief with M-Stim spinal gating, potentially reducing progression to cLBP. Finally, larger studies with chronic opioid users could assess if a multimodal M-Stim device is superior to an implanted stimulator, saving money and reducing morbidity.

## Limitations

As the first investigation of a single non-invasive device to replace opioids acutely and reduce chronic pain, the ability to accurately power all outcomes was limited by a lack of consensus for meaningful reduction of exposure risk. While the primary outcome of prescribing in the opioid-naïve subjects did not reach statistical significance, opioid consumption metrics in this group and use day metrics for those with BMI ≥30 were statistically significant.

The decision to eliminate a potential bias from on-site prescribing required capturing real-world opioid use behaviors. The data collection algorithm was unverifiable through public records and may have over- or undercounted prescribing. Within-patient reporting, however, was highly consistent, and over 95% of entries reported a source. One M-Stim participant was counted as “new prescription” by protocol, but 30/32 of the subject's diaries before and after reported “to me for another event.” If misattributed, M-Stim would have significantly reduced prescribing compared with TENS. The fact that one subject could change significance underscores the need for more subjects and also suggests the DOSE tool should add verification if entries differ from previous trends.

Our choice of a chiropractic office could also have biased enrollment toward opioid-avoidant participants. One study of Vermont LBP health claims found that 22% of chiropractic and 35% of osteopathic patients filled opioid prescriptions over a year. In their cohort, however, the chiropractic users were significantly younger with less disability (83), and the osteopathic patients described resembled our population, of whom 51% had taken or were taking opioids for LBP.

The chiropractic environment may encourage acceptance of non-pharmaceutical and physical treatments. The FDA defines intractable pain as having tried three or more interventions without relief. By this definition, all but two participants had intractable pain, with over a quarter in each arm trying five or more non-pharmacologic interventions. Other environments may be less enthusiastic about applying a device rather than receiving a prescription.

We anticipated regression modeling in the case of unequal groups (e.g., BMI) and realized that providing any intervention for pain could reduce opioid prescribing. Hence, the comparison of prescribing against a national model in addition to against the control was added. We did not apply a Bonferroni adjustment, as the comparison was theoretically grounded in the construct of comparing two similar pain relief modalities (electricity vs. vibration) against a new device with additional opioid-sparing psychosocial considerations (multimodal options). Most importantly, the results favoring M-Stim to reduce opioid use were consistent across multiple analyses, only lacking significance due to being underpowered against a known pain relief device (84).

## Conclusion

Among chiropractic patients with moderate-to-severe LBP, added use of a multimodal M-Stim device in the opioid-naïve subjects significantly reduced factors associated with OUD compared with TENS and reduced use days for those with BMI ≥30. Our results affirm guidelines recommending non-pharmacologic, multimodal LBP treatments as first line to mitigate opioid over-prescribing and signal a potential new approach for cLBP opioid reduction. M-Stim's multimodal approach combines therapies for local, neuromodulatory, and central pain, embodying best practices to reduce psychosocial factors linked to seeking opioids for pain. If verified in other environments, incorporating multimodal devices into routine care could reduce new opioid dependence and reduce excess prescribing. Finally, future studies should collect data on other opioid sources beyond point-of-care prescribing to get a full picture of opioid use.

## Data Availability

The raw data supporting the conclusions of this article will be made available by the authors, without undue reservation.

## In Memoriam

We acknowledge Louise Lawson, PhD, for her passion and statistical expertise supporting this research years before devices as opioid alternatives were potentially fundable. Lawson passed away on 16 April 2020.
