# Supplementary material for: Preventing opioid prescribing for low back pain using multimodal mechanical stimulation vs. TENS: a randomized-controlled trial
Source: Front Pain Res (Lausanne). 2025 Jul 10;6:1612572. doi: 10.3389/fpain.2025.1612572 (PMC12287057; doi:10.3389/fpain.2025.1612572)
Supplement: Supplementary file 1 [file Datasheet1.docx]

Supplemental Table 1

SPIRIT Table: eProtocol for M-Stim for Low Back Pain Opioid Reduction

| Administrative | Description |
| --- | --- |
| Title | Multimodal Mechanical Stimulation to Reduce Opioids for Low Back Pain: A Randomized Clinical Trial  An Outpatient, Double-blinded 3- or 6-Month Randomized Trial Comparing the Impact of DuoTherm™ Mechanical Stimulation (M-Stim) Versus TENS on Opioid Prescribing and Use for Low Back Pain |
| Trial Registration | ClinicalTrials.gov NCT04491175 |
| Protocol Version | NIH funded protocol submitted 5/29/2019 to FDA Q191165  IRB Protocol Submission Date: 8/10/2019  Protocol Amendment Number: 4  Authors: A.B.,K.S.,L.L.  Revision Chronology:   1. 8/10/2019 Original   [COVID delay]   1. 4/21/2022 FDA recommended modifications from Q191165 incorporated to protocol: increased sample size to 60 opioid naïve and 100 chronic (with 6 months followup); Replaced Sham with LG Smart TENS and added a blinding assessment; Outcome measures of PROMIS Pain Interference , Pain Intensity, Depression to be measured weekly the first month. 2. 6/8/2022 – Final survey design reduced daily diary reporting to 1 month due to budget constraints of longer followup. Depression and Catastrophizing only as baseline; weekly NRS Pain Interference (3) PROMIS Pain Interference (2) and Pain Intensity collected until 3 months for all; Monthly surveys added past month NRS, Blinding assessment for additional 3 months for those started in chronic strata.   Added “source” for each opioid use case to DOSE tool collecting opioid formulation, dose, and number of pills.   1. 5/5/23; Added “Do you think you will need surgery” and “do you think you will need epidural” questions, and “did you get surgery” and “did you get an epidural” to monthly. 2. 7/13/2023 New Location added |
| Funding | National Institute on Drug Abuse, R44DA049631, R44DA058952 |
| Roles and Responsibilities | Principal Investigator: Amy Baxter MD, responsible for study design, overall project oversight, and compliance. Study Coordinator: Jena Slaski, responsible for daily operation, protocol adherence and patient coordination at Kaizo Health Centers. Lindsey Cohen assisted Dr. Baxter with initial study design, ongoing recruitment recommendations, pain measure decision-making, study flow creation. M. Louise Lawson PhD (Deceased), responsible for initial power analysis, statistical support for study design, revisions after FDA. Dr. Kevin Swartout PhD, responsible for programming of data collection instruments, ongoing support of data collection tool and dataset, database consolidation; Jessica Allia Williams PhD, responsible for statistical analysis of completed dataset. All apart from Dr. Lawson reviewed the manuscript. |
| **Introduction** | Low back pain (LBP) affects nearly 40% of adults yearly, with 60% affected in their lifetimes and 40% progressing from acute to chronic pain. It is the single biggest contributor to worldwide disability. The opioid epidemic, largely driven by prescriptions for pain, has created an urgent need for effective non-pharmacological pain treatments. Existing options such as TENS devices may provide acute relief, but are not covered as an opioid substitute. A multimodal approach, combining mechanical stimulation, heat, cold, and acupressure, holds promise for reducing both acute and chronic pain and opioid prescribing. |
| Background and rationale for the study | Between 25 and 50% of patients presenting with acute or exacerbated chronic LBP (a/cLBP) receive prescriptions for opioids. ALBP progresses to chronic in 40% of those with moderate-to-severe pain (4 out of 10 on a 0 – 10 Numeric Rating Scale). Opioid pain relief is no better than placebo at 6 weeks after presentation, but increases the risk of cLBP and persistent use (5% of opioid naïve). Providing a multimodal low back pain device could reduce opioid prescribing or use compared to TENS; providing any device could reduce opioid prescribing and use compared to contemporaneous prescribing practices, leading to a new FDA indication if 50% relief or 30% improvement compared to an active control. |
| Objectives | **Primary Objective, Opioid Reduction:** To determine whether the use of a multimodal neuromodulatory mechanical stimulation (M-Stim) device (DuoTherm) can reduce prescribing of opioid medications 30% compared to TENS in the opioid-naive with moderate to severe back pain. **Secondary Objectives:** To determine whether multimodal M-Stim reduces the days of use and use after 7 days in the opioid-naïve, and versus contemporaneous national prescribing, and using MME for those with chronic opioid use for LBP.  **Primary Objective, CLBP Reduction:** To determine whether M-Stim reduces disability compared to TENS over 3 or 5 months using PROMIS Pain Interference. Secondary Objectives: To determine whether M-Stim is noninferior compared to TENS for acute pain using a NRS after 30 minutes and 10 days; to compare NRS after 3 and 6 months; to evaluate the progression of acute to chronic pain over 3 months for those with aLBP. |
| Study Design | A randomized, double-blind controlled trial with 3 m followup for opioid outcomes, and 3-aLPB or 6- month (cLPB) follow-up for pain outcomes. |
| Study Setting | The study will be conducted at mixed physical therapy/chiropractic practices in Washington, DC, Maryland, and Virginia, which offer demographic and economic diversity. These clinics provide an ideal environment, with frequent follow-ups and support for alternative therapies but no on-site access to opioid prescribers. |
| Eligibility Criteria | Inclusion Criteria: - Male or female adults aged 18-90 with diagnosed acute or chronic low back pain  - Self report moderate to severe LBP of 4 or greater out of 10 on a 0-10 Numeric Rating Scale.  - Acute: pain duration <3 months without opioid use for low back pain  - Chronic: Pain duration >=3 months with or without chronic opioid use - Capacity to understand risks and benefits (informed consent).  - Smartphone for Qualtrics web-based platform use Exclusion Criteria:  - Radicular pain likely reflecting a surgical or mechanical problem  - Inability to apply or use the device, BMI (>30 with prototype, >50 with new device) or staff evaluation of fit with appropriate use (sitting or standing)  - Sensitivity to cold or vibration (e.g Raynaud’s or Sickle Cell Disease)  - Diabetic neuropathy rendering a patient unable to determine if the device is too hot  - New neurologic deficits, skin lesions over the low back area  - A contraindication to any medication for pain management that would impact analgesic use record  - Pacemaker |
| Intervention | The experimental arm is a novel multimodal low back pain relief device incorporating 8 harmonic vibration patterns of mechanical stimulation (M-Stim), and optional heat, cold, and pressure delivered through a sculpted metal plate attached with a belt and controlled by buttons on the belt. The active control is a prescription 4-lead 8-channel TENS unit (LG Smart). Participants will be instructed to use the device for a minimum of 30 minutes a day. Both M-Stim and LG Smart TENS groups will receive a standard pain management regimen typically used for low back pain standardized across all clinicians participating in the study. Participants will keep the devices and be instructed to use them for pain as needed during the duration of the study. |
| Outcomes | The primary study question was whether dispensing a multimodal pain relief device at presentation for treatment with moderate to severe pain reduces opioid use. We hypothesized that providing a multimodal M-Stim device would reduce prescribing by 30% in the opioid naïve. Secondary outcomes were MME (Milligram Morphine Equivalents) between the first 14 days and the last 14 days of daily diary recordings for those who took opioids, and reduced opioid prescribing compared to national rates compared to TENS.  **Secondary outcomes** included risk factors of prolonged opioid use (POU) in the opioid naive, including days of use, continued opioid use after 7 days, and total MME in the opioid naïve, and prescribing compared to a contemporaneous national LBP population (25%). |
| Participant Timeline | \|  \| Initial Visit \| Daily x 28 days \| Weekly for 3 months \| Monthly for 3 months \| Monthly 4 - 6 months \| \| --- \| --- \| --- \| --- \| --- \| --- \| \| Consent, Enrollment, Randomization \| Albp, clbp \|  \|  \|  \|  \| \| Minimum Dataset, Pain Catastrophizing Sullivan, PROMIS Depression, Physical Function \| Albp, clbp \|  \|  \|  \|  \| \| NRS (at that moment & past 24 hours) \| Albp, clbp \| ALBP, CLBP \| Albp, clbp \| Albp, clbp \| clbp \| \| NRS after 30 minutes \| Albp, clbp \|  \|  \|  \|  \| \| NRS Past week \|  \|  \| Albp, clbp \| Albp, clbp \| clbp \| \| NRS Past month \|  \|  \|  \| Albp, clbp \| CLBP \| \| PROMIS Pain Intensity, Pain Interference \| Albp, clbp \|  \| Albp, clbp \| Albp, clbp \| CLBP \| \| Dose, Opioid formulation, number Source (DOSE tool), other analgesics \| Albp, clbp \| ALBP, CLBP \| ALBP, CLBP \| ALBP, CLBP \| CLBP \| \| Device usage \|  \| ALBP, CLBP \| ALBP, CLBP \| ALBP, CLBP \| CLBP \| |
| Sample Size | To estimate the M-Stim intervention effect size, a retrospective study of 59 cLBP patients on chronic opioids (100% prolonged opioid use) reported a 28% reduction in MME after spinal stimulator implantation,[40] reducing MME 26.2(SD32.8) compared to 5.8(SD34.2) for those with conventional treatment (effect size 0.60. A priori, we would be able to detect an effect size of 1 (meaning a difference of 1 standard deviation) with 94% power and alpha of .03 using a Wilcoxon Mann Whitney test with 30 in each group. With this effect size for a two-sided significance of 0.05, 23 participants per group would detect a 30% prescribing reduction. For cLBP, 50 participants on chronic opioids per treatment group would provide 85.4% power to detect a 30% reduction in MME. We planned to recruit 60 participants who had never taken opioids for their back pain, and 100 cLBP for disability outcomes with or without chronic opioid use for cLBP. |
| Recruitment | Patients were recruited from Kaizo Health Centers in Landover and Fairfax, through direct referrals, flyers, and digital content like social posts and emails targeting local populations. Flyers were also distributed at three local pain clinics. |
| Randomization | After eligibility screening and informed consent, a 4-digit participant ID and the intervention allocation were [randomly assigned](https://www.qualtrics.com/support/survey-platform/survey-module/survey-flow/standard-elements/randomizer/)  by the survey software to either the DuoTherm or LG Smart TENS group within acute and chronic arms. A copy of the informed consent was emailed to the participant. |
| Blinding (masking) | Participants were blinded to which device powered the study hypothesis, and blinded to their assigned intervention until after documenting initial NRS pain intensity. Success of blinding the study device (M-Stim) versus the active control (TENS) was tested at months 3-6 with prompts, “select which group you think you were in (control or treatment)” and “How confident are you (0 = Least Confident ... 100 = Most Confident).”  The protocol statistician and study coordinator knew the intervention assignments during enrollment but did not conduct statistical analysis. The PI and treating chiropractor were blinded to allocation during enrollment, with the PI accessing data only after study completion. The analyzing statistician was blinded to device assignment until completion of data cleaning and initial analysis. |
| Data Collection Methods | Site coordinators entered identification and contact information after informed consent on a tablet, then recorded weight, height, and duration to confirm acute or chronic follow-up duration, then participants took the tablet to record pain intensity on a 0-10 NRS now and past 24 hours. While using the device for 30 minutes, participants completed 7-day pain intensity NRS, NIH Back Pain Research Minimum Dataset, including PROMIS measures of Physical Function, Pain Interference, Depression, and Catastrophizing via the Sullivan Catastrophizing scale, Injury Mechanism, prior treatments, exercise and athletic status.  Participants were prompted to record analgesic use, pain, and device use daily for a month on the web-based platform, then weekly, with the monthly surveys adding Pain Interference. All surveys asked about analgesic use. Due to the anticipated heterogeneity of opioid formulations, a skip-logic data collection instrument algorithm was created with robust options for Dose per pill (34), Opioid formulations, e.g. hydrocodone, hydromorphone etc. (15) and the Source (DOSE tool, Supplement 2). Sources included “prescribed to me for this event”, “prescribed to me for another event”, “given to me by a family member”, “given to me by a friend”, “given to me by an acquaintance or stranger”, or “purchased from someone without a prescription”. These were converted to a total milligram morphine equivalent (MME) for analysis.  Participants recorded when they used their device, duration of use, and any additional pain relief modalities (exercise, stretching, bath, hot tub, therapy, massage). Patients were asked to record any thermal use or therapy cycle or channel as appropriate to their device.  Participants verbally endorsing pain for less than three months (acute) were entered into a 3 month follow-up program, or 6 months (chronic) for those for whom back pain had been an ongoing problem for more than 3 months. |
| Statistical Methods | Statistical analysis was performed with masked intervention allocation by a statistician new to the project to determine differences in primary and secondary outcomes.  An intention-to-treat analysis included all participants meeting inclusion criteria. Summary statistics (means, standard deviations, proportions) were calculated using T-tests and Chi-squared tests. Opioid prescribing differences were calculated with raw percent of change and relative risks. Diary entries and item-level data were analyzed for missing data to assess the need for imputation. Relative risks were calculated for MME and opioid use days between intervention groups, with paired T-tests used to compare first two and last two weeks of MME in those who used opioids during the study. A linear regression assessed BMI's interaction with opioid use, calculating marginal effects with standard errors via the Delta Method. Data analysis was performed using STATANow/SE 18.5 and online MedCalc. |
| Data Monitoring | Data collection was conducted through an app that tracked daily pain, opioid use and device usage. Monitoring included weekly enrollment review by the protocol statistician to assess comprehension of the data collection tool. Adverse events were reported as per protocol.  Obtained data was stored on password-protected network drives made accessible solely to the listed investigators. Data analysis will only be conducted in secure rooms with restricted access. These rooms will only be accessible to investigators. Patient samples will not be obtained. The Institutional Review Board reserves the right to audit any research files to assure the quality of any data used in this research |
| Safety/Harm | Q191165/S001 FDA ruled Non-significant Risk 18-Sept 2019. Risks are minimal, similar to those associated with a handheld massager or standard heat/cold therapy. Patients will be screened for sensitivity to cold or vibration. Any adverse events will be reported to the IRB and NIH within 24 hours. |
| Auditing | Periodic auditing will be conducted by the Institutional Review Board (IRB) to ensure compliance with protocol and safety regulations, and by site visits by the PI. |
| Ethics Approval | This trial has received ethics approval from the Kaizo Clinical Research Institute IRB and is registered under NCT04491175. |
| Protocol Amendments | Revision Chronology:   1. 8/10/2019 Original   [COVID delay]   1. 4/21/2022 Incorporated FDA recommended modifications from Q191165 incorporated to protocol: increased sample size to 60 opioid naïve acute and 100 chronic (with 6 months followup); Replaced Sham with LG Smart TENS active control with 8 pre-programmed patterns to correspond with 8 pre-set DuoTherm therapy cycles and added blinding assessment. Outcome measures of PROMIS Pain Interference , Pain Intensity, Depression to be measured weekly the first month. We extended the follow-up from one week for acute pain to 3 months for acute pain and 6 months for chronic pain. 2. 6/8/2022 – Final survey design reduced daily diary reporting to 1 month due to budget constraints of longer follow-up. Depression and Catastrophizing only as baseline; weekly NRS Pain Interference (3) PROMIS Pain Interference (2) and Pain Intensity collected until 3 months for all; “Past month NRS” added to monthly surveys added past month NRS, Blinding assessment for additional 3 months for those started in chronic strata.   Added “source” for each opioid use case to DOSE tool collecting opioid formulation, dose, and number of pills.  We intended to use evaluate change in MME over time for chronic opioid users. The FDA recommended requiring participants on opioids maintain dosage and frequency. As we did not have access to prescribing records, and opioid reduction was a desired outcome measure, we evaluated opioid use for the first two weeks versus the last two of those endorsing prior opioid use. We extended the baseline and follow-up Pain Intensity measurements to one month rather than one week.   1. 5/5/23; Added “Do you think you will need surgery” and “do you think you will need epidural” questions, and “did you get surgery” and “did you get an epidural” to monthly. 2. 7/13/2023 New Location added   **Stratification by chronicity versus opioid use status:** enrollment strata were based on verbal endorsement of duration and no acute opioid use for back pain. Initial evaluation of registration data after enrollment was complete revealed 16 of 60 in the “acute” strata reported a duration longer than three months, and half in each chronicity strata no prior opioids for LBP. In consultation with Clinical Trials.gov, we pooled the data and maintained the acute/opioid naïve registry for opioid use outcomes NCT04491175, and chronic/mixed for pain outcomes NCT04494698. |
| Informed Consent | Informed consent will be obtained from each participant after screening. Participants will be informed about the voluntary nature of the study, with a HIPAA form for confidentiality. Consent will be documented via selecting ‘continue’ on the digital consent form. |
| Confidentiality | Patient data will be stored on password-protected network drives. All data will be de-identified using a unique study ID for each participant. Confidential records will be stored in secure, restricted-access rooms. |
| Declaration of interests | The principal investigator has financial conflict of interest from design and research into the DuoTherm device, and creating a company to develop the device with NIH SBIR funding. The collaborators declare no financial or personal conflicts of interest related to this study. |
| Dissemination Policy | Results will be disseminated through peer-reviewed journals, conferences, and possibly through NIH publications, ensuring that both positive and negative outcomes are reported. |
| Ancillary and Post-Trial Care | Patients will receive standard care post-trial and may keep their devices if found beneficial. Participants will be provided referrals for ongoing care as needed. |
| Publication Plan | High impact journal, with presentation at the IASP or similar pain-related conference. |
