# Supplementary material for: Preventing opioid prescribing for low back pain using multimodal mechanical stimulation vs. TENS: a randomized-controlled trial
Source: Front Pain Res (Lausanne). 2025 Jul 10;6:1612572. doi: 10.3389/fpain.2025.1612572 (PMC12287057; doi:10.3389/fpain.2025.1612572)
Supplement: Supplementary file 2 [file Datasheet2.docx]

**Supplement 2: DOSE Tool**

Due to the heterogeneity of potential outpatient opioid formulations, a data collection instrument algorithm was created to collect Dose, Opioid, and the Source of different brands in circulation without relying on free text. Using skip-logic prompts programmed into Qualtrics (Provo, Utah, USA), during registration participants were asked “What short acting opioid medications have you taken for your pain” and with daily and weekly surveys “…since your last survey? (Please select all that apply)”.

The potential choices included 8 opioid formulations (e.g. Hydrocodone/APAP (acetaminophen)) followed by all potential brands currently on the market (e.g. “Hydrocodone/APAP (acetaminophen) [ex: Lorcet, Lortab, Norco, Vicodin, Zydone, Generic]) or “I did not take any short-acting opioids”. The next prompt asked which specific brand was taken, giving choices of all dose combinations for that brand and how many pills were taken. The final prompt of “This medication was …” probed for source with 6 possible choices: “prescribed to me for this event”, “prescribed to me for another event”, “given to me by a family member”, “given to me by a friend”, “given to me by an acquaintance or stranger”, or “purchased from someone without a prescription”. The same question was asked for six long-acting opioids. Opioid prescribing outcomes included any response by any participant that included “to me for this event”, while opioid use included any recorded opioids. To compare all quantitative opioid responses, MME were calculated using the Heath and Human Services conversion tables.[1]


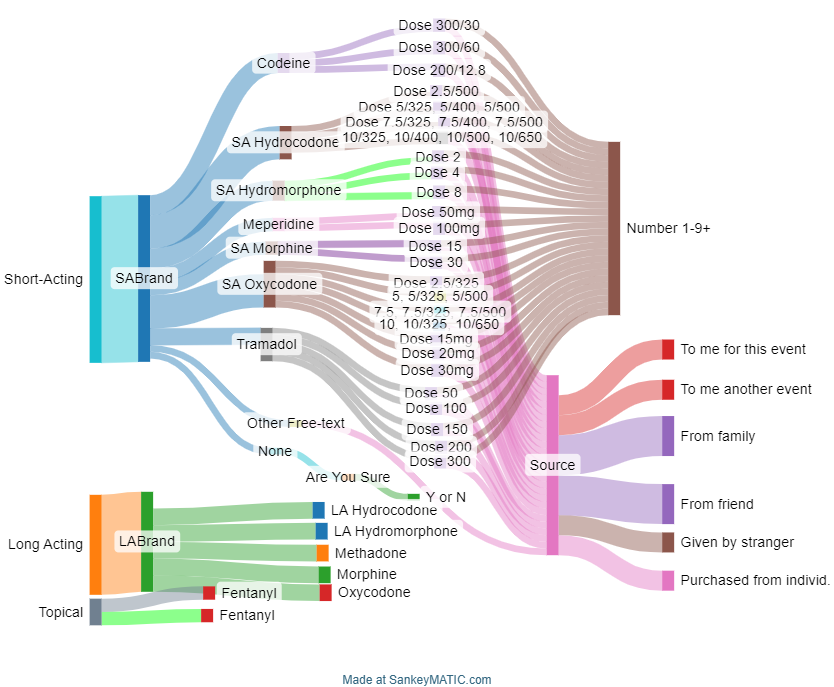


The initial DOSE tool will be modified in future iterations based on study outcomes.

The category of “Other Short Acting [ex: Dihydromorphine, Nicomorphine, Oxycodone with Aspirin or Ibuprofen, Oxymorphone]” will be removed and the answers will not be considered in the opioid use, as the subjects appeared to be responding to the Aspirin or Ibuprofen prompt, not realizing it was a combination product.

**The next iteration of the tool will allow for overwriting when backspaces are applied.**

Fentanyl patch use or medication assisted treatment (buprenorphine).

**Future iterations will not allow more than 1 week recordings, and will allow for free-texting an amount if more than 9 pills.**

When an opioid has both a long and a short acting form, and the participant entered the same name, dose, pill number and source under both SA and LA, **future iterations will include logic to verify and eliminate one or the other.**
