# Supplementary material for: Preventing opioid prescribing for low back pain using multimodal mechanical stimulation vs. TENS: a randomized-controlled trial
Source: Front Pain Res (Lausanne). 2025 Jul 10;6:1612572. doi: 10.3389/fpain.2025.1612572 (PMC12287057; doi:10.3389/fpain.2025.1612572)
Supplement: Supplementary file 3 [file Datasheet3.docx]

Supplement 3: Informed Consent and Data Collection

Registration Survey (Acute)

Start of Block: Consent

**Participant Registration Survey (${e://Field/Group} Pain)**

Which location is enrolling the patient in the study?

- Fairfax, VA
- Landover, MD

**MOVE TO THE NEXT PAGE
 THEN HAND TABLET TO PARTICIPANT**

[Page Break]

**INFORMED CONSENT FORM**
    
**Addressing Opioid Use Disorder with an External Multimodal Neuromodulation Device: Clinical Evaluation for Opioid-Sparing in Acute Low Back Pain**

 You have been invited to take part in this study. It is important that you read and understand the info below before you agree to participate. Your relationship with the clinic will not change if you decide not to be in the study. Please ask questions about what you do not understand before agreeing to take part.

 **Purpose of the Study**
 The purpose of this study is to determine whether providing a stimulation device to patients with acute low back pain (LBP) reduces pain and opioid use.

**Procedures**
 If you agree to be in this study, we will ask you to do the following things: After completing the initial paperwork, you will rate your pain and complete background paperwork. Next, the computer program will determine your group assignment to one of two neuromodulation devices designed for daily home use using either mechanical or electrical stimulation both with multiple therapy cycles.

 After your group assignment, you will receive a standard pain management regimen typically used for low back pain. Regardless of group assignment, you will be instructed on how to use the data collection system via an online instruction video. We are collecting information on opioid use. Therefore, we will ask you to complete the surveys on how much medication you are taking, including if there are different kinds that may be from different sources and medication you have taken prior to getting a prescription. You will be prompted by the **text message with a link** to record the following information on the website: Daily for one month – use of pain medication and pain relief (Device, exercise, stretching, bath, hot tub, therapy, massage, etc.). Weekly – how pain interferes with movement and how intense it is Months 1, 2, 3– changes in function and your mood Your participation will consist of an hour for paperwork/instruction today and then a quick 2-minutes survey daily for a month. Each week there will be a 15-minute questionnaire for the full three months of the study.

[Enrollment Chart from SPIRIT Table]

***Risks* and *Benefits* of being in the study**

 **Possible Risks:** The risks of the device should be no greater than the risks of a hand-held massager, electrical stimulator, or hot or cold pack depending on which group you’re in. The possibility exists that the focus on pain assessment will draw attention to the LBP and could increase your pain. You will be given your own device so risk of transmitting infection will be minimal. In previous research, patients with LBP supported that the use of the device is helpful; no patient has stated that it increases pain or causes other problems. The risk of receiving no intervention is the same risk which is standard of care for patients being treated for LBP throughout the country. Electrical stimulation is already a standard of care and found safe for LBP. Cold and heat are already a standard of care in home remedies for LBP – the possibility of increased sensitivity to cold exists, but you choose whether or not to use the cold portion if you’re in this group. Patients receiving cold therapy will not be exposed to enough cold to result in frostbite. Likewise, if you do not want heat, you do not have to use heat with your device, or if you do not want electricity you don’t need to use it.

 **Benefits:** This study will determine whether different kinds of low back stimulation for patients with LBP will reduce opioid use. Achieving our aims implies, you may reduce your use of pain medication or not need to start opioids. The results of the study may also be translated to other musculoskeletal complaints where inflammation, pain and stiffness are concerns that decrease quality of life. If the stimulation device increases compliance with medical care, it may diminish pain, stress and reduce opioid use, which provides a significant benefit to you. Conceptually it will challenge the pharmaceutical focus of current pain strategies, changing practice in the field of pain relief and rehabilitation. If there are lower unused opioids in circulation, this is also an important addition to combatting opioid use disorder.

**Confidentiality**
 All info you reveal in this study will be kept confidential. Your records will be assigned a random number instead of using your name. Documents will be stored in a locked cabinet and electronic files will be kept on a secure computer. Only members of the research team will have access to these records. Three years after the study, we will shred all papers. When the study is published your name will not be included. Your records may be looked at by the Kaizo Clinical Research Institute IRB, Department of Health and Human Development, the Food and Drug Administration, and state and federal agencies.

 **Compensation**
 You will be compensated for each of the questionnaires you complete according to the following schedule. Weeks 1 and 2: $1 a day for every time you complete the daily form. If you complete 7 days in a row you will earn a $3 bonus for hitting the streak. Weeks 3 and 4: $2 a day for every time you complete the daily form. If you complete 7 days in a row you will earn a $6 bonus for hitting the streak. Months 2 and 3: $10 every time you complete a weekly survey. If you complete four weekly surveys in a row you will earn a $10 bonus for hitting the streak. This will total a maximum of $160 you can earn. You will be paid with an Amazon gift card at the end of each month. You must complete 80% of the data in weeks 1 and 2 to continue to weeks 3 and 4. If you complete 80% of the data in the first month you get to keep the assigned device. If you do not, we will ask for you to return the device and you will forfeit the money earned during this first month.

**Injury or Illness**
 If you say YES to participate, then your consent in this document does not waive any of your legal rights. However, in the event you are injured or become ill as a result of participating in this study, neither Kaizo Health, Kaizo Clinical Research Institute, Kaizo Clinical Research Institute IRB, or any other researchers are able to give you any money, insurance coverage, free medical care, or any other compensation from such injury. In the event that you suffer injury as a result of participation in any research project, you may contact Dr. Jay Greenstein at (301) 518-1006 or Dr. Barton Bishop the current IRB chair at (240) 766-0300 x835 at Kaizo Clinical Research Institute, who will be glad to review the matter with you.

 **Voluntary Nature of the Study:**
 Taking part in this study is completely voluntary. You may withdraw and stop participating at any time. If at any time, you wish to withdraw from the study please let any member of the research staff know. 

 **Contacts and Questions**
 If you have any questions, you can call Jena Slaski (240) 766-0300x838. If you have concerns about your rights as a research participant, you can contact Kaizo Clinical Research Institute IRB Chair, Barton Bishop at (240) 766-0300x835.

 **Statement of Consent**

 **I have read the info above. I have had the chance to ask questions and have them answered. Tapping “next” below signifies that I agree to participate in the study and have been given a copy of the consent form.**

[Page Break]

**PLEASE HAND THE TABLET TO THE STAFF MEMBER.**

End of Block: Consent

Start of Block: Registration Survey

**PLEASE RECORD THE FOLLOWING INFORMATION IN THE PARTICIPANT'S FILE**

Participant ID:**${e://Field/LoginID}**

Display This Question:

If Condition = 1

Participant Condition:  **DT**

Display This Question:

If Condition = 2

Participant Condition:  **LG**

[Page Break]

Participant Contact Information and Body Measurements (completed by staff member)

- First Name __________________________________________________
- Last Name __________________________________________________
- Email Address __________________________________________________
- Cell phone number (1-555-555-5555) __________________________________________________
- Patient Height (in inches) __________________________________________________
- Patient Weight (in lbs) __________________________________________________

How long has the patient experienced ongoing back pain? (If greater than 3 months, switch to the chronic survey.)

- 1-2 days
- 3-4 days
- 5 days-1 week
- 1-2 weeks
- 3 weeks-1 month
- 1-2 months
- 2-3 months

[Page Break]
**MOVE TO THE NEXT PAGE THEN HAND TABLET TO PARTICIPANT**

[Page Break]

| 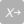 |
| --- |

Please Rate Your Current and Recent Pain Using the Sliding Scales Below   (0 = No Pain......10 = Worst Possible Pain)

|  | 0 | 1 | 2 | 3 | 4 | 5 | 6 | 7 | 8 | 9 | 10 |
| --- | --- | --- | --- | --- | --- | --- | --- | --- | --- | --- | --- |
| Pain Now |  |  |  |  |  |  |  |  |  |  |  |
| Pain over the past 24 hours |  |  |  |  |  |  |  |  |  |  |  |

At this time, how likely do you think it is you will need surgery for your low back pain issue?

- Extremely unlikely
- Unlikely
- Neither likely or unlikely
- Likely
- Extremely likely

At this time, how likely do you think it is you will need an epidural injection for your low back pain issue?

- Extremely unlikely
- Unlikely
- Neither likely or unlikely
- Likely
- Extremely likely

[Page Break]

**PLEASE HAND THE TABLET TO THE STAFF MEMBER.**
 **THEY WILL SHOW YOU THE INSTRUCTIONAL VIDEO**
**THEN BEGIN THE DEVICE THERAPY**

[Page Break]

Time device therapy started

________________________________________________________________

[Page Break]

**MOVE TO THE NEXT PAGE THEN HAND TABLET TO PARTICIPANT**

[Page Break]

How long has low back pain been an ongoing problem for you?

- Less than 1 month
- 1-3 months
- 3-6 months
- 6 months-1 year
- 1-5 years
- More than 5 years

How often has low back pain been an ongoing problem for you over the past 3 months?

- Every day or nearly every day in the past 3 months
- At least half the days in the past 3 months
- Fewer than half the days in the past 3 months

How many days ago did your current episode of low back pain start?

- 1-2 days
- 3-4 days
- 5-6 days
- 1 week
- 2 weeks
- More than 2 weeks

Is your low back pain more severe than pain in other parts of your body?

- Yes
- No
- Not sure

How do you believe your back pain started? (select all that apply)

- Muscle pull
- Sitting/strain
- Bone injury
- Lifting
- Fall
- Work overuse
- Penetrating injury
- Motor vehicle collision
- Other (Please specify) __________________________________________________
- I don't know

| In the past 7 days… | Had no pain | | Mild | | Moderate | | Severe | | Very severe | |
| --- | --- | --- | --- | --- | --- | --- | --- | --- | --- | --- |
| How intense was your pain at its worst? |  | |  | |  | |  | |  | |
| How intense was your average pain? |  | |  | |  | |  | |  | |
| In the past 7 days... | | Not at all | | A little bit | | Somewhat | | Quite a bit | | Very much |
| How much did pain interfere with your day-to-day activities? | |  | |  | |  | |  | |  |
| How much did pain interfere with work around the home? | |  | |  | |  | |  | |  |
| How much did pain interfere with your ability to participate in social activities? | |  | |  | |  | |  | |  |
| How much did pain interfere with your household chores? | |  | |  | |  | |  | |  |
| How much did pain interfere with the things you usually do for fun? | |  | |  | |  | |  | |  |
| How much did pain interfere eith your enjoyment of social activities? | |  | |  | |  | |  | |  |
| How much did pain interfere with your enjoyment of life? | |  | |  | |  | |  | |  |
| How much did pain interfere with your family life? | |  | |  | |  | |  | |  |

Physical Function - How much difficulty do you experience with the following activities?

|  | No difficulty | A little | Some difficulty | Much | Unable to do |
| --- | --- | --- | --- | --- | --- |
| Chores such as vacuuming or yard work |  |  |  |  |  |
| Going up and down stairs at a normal pace |  |  |  |  |  |
| Go for a walk of at least 15 minutes |  |  |  |  |  |
| Run errands and shop |  |  |  |  |  |

| When I'm in pain... | Not at all | Slightly | Moderately | To a great degree | All the time |
| --- | --- | --- | --- | --- | --- |
| I worry all the time about whether the pain will end |  |  |  |  |  |
| I feel I can't go on |  |  |  |  |  |
| It's terrible and I think it's never going to get better |  |  |  |  |  |
| It's awful and I feel that it overwhelms me |  |  |  |  |  |
| I feel I can't stand it anymore |  |  |  |  |  |
| I become afraid that the pain will get worse |  |  |  |  |  |
| I keep thinking of other painful events |  |  |  |  |  |
| I anxiously want the pain to go away |  |  |  |  |  |
| I can't seem to keep it out of my mind |  |  |  |  |  |
| I keep thinking of how much it hurts |  |  |  |  |  |
| I keep thinking of how badly I want the pain to stop |  |  |  |  |  |
| There's nothing I can do to reduce the intensity of the pain |  |  |  |  |  |
| I wonder whether something serious may happen |  |  |  |  |  |

In the past 7 days...

|  | Never | Rarely | Sometimes | Often | Always |
| --- | --- | --- | --- | --- | --- |
| I felt worthless |  |  |  |  |  |
| I felt helpless |  |  |  |  |  |
| I felt depressed |  |  |  |  |  |
| I felt hopeless |  |  |  |  |  |

Have you ever had a low back operation?

- Yes, once
- Yes, more than once
- No

Display This Question:

If Have you ever had a low back operation? = Yes, once

Or Have you ever had a low back operation? = Yes, more than once

Did any of your back operations involve a spinal fusion?

- Yes
- No
- Not sure

Have you been off work or unemployed for 1 month or more due to low-back pain?

- Yes
- No
- Does not apply

Have you filed or been awarded a worker’s compensation claim related to your back problem?

- Yes
- No
- Does not apply

Are you involved in a lawsuit or legal claim related to your back problem?

- Yes
- No
- Not sure

Have you ever applied for, or received, disability insurance for your pain condition?

- Yes
- No

[Page Break]

Have you used any of the following treatment for your low back pain? (check all that apply)

- Over the counter medications
- Exercise therapy
- Injections (facet, steroid)
- Vibration therapy
- Cannabis/Marijuana
- Cold therapy
- Heat therapy
- Acupressure therapy
- Massage therapy
- TENS unit therapy
- Psychological counseling, such as Cognitive Behavioral Therapy
- Topical creams (CBD, menthol, etc.) please specify __________________________________________________
- I have not used any of these treatments

[Page Break]

How often do you exercise?

- No exercise
- 1-2 times/week
- 3+ times/week
- Competitive athlete

Display This Question:

If How often do you exercise? = 1-2 times/week

Or How often do you exercise? = 3+ times/week

Or How often do you exercise? = Competitive athlete

How vigorously do you exercise?

- Low impact (walking)
- Moderate impact
- Intense

[Page Break]

How would you describe your cigaratte smoking?

- Never smoked
- Current smoker
- Used to smoke, but have now quit

In the past year...

|  | Never | Rarely | Sometimes | Often | Always |
| --- | --- | --- | --- | --- | --- |
| Have you been drunk or used drugs more than you meant to? |  |  |  |  |  |
| Have you felt you wanted or needed to cut down on your drinking or drug use? |  |  |  |  |  |

[Page Break]

What short acting medications have you taken for your pain in the past? (Please select all that apply)

- Codeine [ex: Tylenol w/ codeine, Neurofen, T#3]
- Hydrocodone/APAP (acetaminophen) [ex: Lorcet, Lortab, Norco, Vicodin, Zydone, Generic]
- Hydromorphone [ex: Dilaudid, Palladone]
- Meperidine (Demerol)
- Short Acting Morphine Sulfates
- Oxycodone [ex: Percocet, Endocet, Tylox, Oxaydo, Roxicodone, Generic]
- Tramadol [ex: Ultram, ConZip]
- Other Short Acting [ex: Dihydromorphine, Nicomorphine, Oxycodone with Aspirin or Ibuprofen, Oxymorphone]
- I have not taken any of these short-acting medications in the past.

What long acting medications have you taken for your pain in the past? (Please select all that apply)

- Hydrocodone [ex: Hysingla, Vantrela, Zohydro]
- Hydromorphone SR [ex: Exalgo, Hydromorph Contin, Jurnista, Palladone SR]
- Methadone [ex: Amidone, Dolophine, Methadose]
- Long Acting Morphine Sulfates [ex: Avinza, MS Contin, Kadian, Oramorph SR]
- Oxycodone HCl [ex: Oxaydo, Oxycontin, Xtampza]
- Fentanyl Patch [ex: Duragesic]
- I have not taken any of these long-acting medications in the past

In the past, have you used any of the following medications for pain? (Please select all that apply)

- Acetaminophen (e.g., Tylenol)
- Gabapentin
- Ibuprofen (e.g., Advil or Motrin)
- Asprin
- Cannabis/Marijuana
- Naproxin Sodium (e.g., Aleeve)
- Another medication not listed on this page (Please specify) __________________________________________________
- I have not taken any of these medications in the past

[Page Break]

Please answer the following demographic questions about yourself.

Date of Birth

________________________________________________________________

Age

________________________________________________________________

Sex at Birth

- Male
- Female
- Unknown
- Intersex

Gender Identity

- Male
- Female
- Unknown
- Other, Please Specify __________________________________________________

Ethnicity

- Hispanic or Latino
- Not Hispanic or Latino
- Unknown
- Not Reported

Race (choose all that apply)

- American Indian or Alaskan Native
- Asian
- Black or African-American
- Native Hawaiian or Other Pacific Islander
- White
- Unknown
- Not Reported

What is the highest level of education you have completed?

- Did not complete Secondary School or Less than High School
- Some Secondary School or High School Education
- High School or Secondary School Degree Complete
- Associate’s or Technical Degree Complete
- College or Baccalaureate Degree Complete
- Doctoral or Postgraduate Education

What is your current employment status?

- Full-time employment
- Not employed
- Part-time Employment

What category best describes your current relationship status?

- Divorced
- Married
- Never married
- Separated
- Widowed
- Domestic Partner

Including yourself, how many people live in your household?

________________________________________________________________

What is your annual household income from all sources?

- Less than $10,000
- $10,000--- $24,999
- $25,000--- $34,999
- $35,000--- $49,999
- $50,000--- $74,999
- $75,000---$99,999
- $100,000--- $149,999
- $150,000--- $199,999
- $200,000 or more
- Prefer not to answer

[Page Break]

**PLEASE HAND THE TABLET BACK TO THE STAFF MEMBER**

[Page Break]

Time device therapy stopped

________________________________________________________________

[Page Break]

**MOVE TO THE NEXT PAGE
 THEN HAND TABLET TO THE PARTICIPANT**

[Page Break]

| 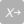 |
| --- |

**How is your pain now that you have used the study device?   (0 = No Pain......10 = Worst Possible Pain)**

|  | 0 | 1 | 2 | 3 | 4 | 5 | 6 | 7 | 8 | 9 | 10 |
| --- | --- | --- | --- | --- | --- | --- | --- | --- | --- | --- | --- |
| 1 |  |  |  |  |  |  |  |  |  |  |  |

[Page Break]

**PLEASE HAND THE TABLET BACK TO THE STAFF MEMBER**

[Page Break]

Initial visit therapy after use of study device:

- Exercises
- Adjustment
- IASTM
- Manual Therapy
- No Other Therapy

End of Block: Registration Survey

Start of Block: Post-Survey Script

**Post-Registration Script**
 Thank you for registering for this study!

 Once I record your survey responses by clicking the button below, you will receive an email with the full consent form you read at the beginning of the survey. 

 As a reminder, this is a 3-month study. You will receive emails and text messages each day for the first 4 weeks, beginning tomorrow evening, with links to complete daily surveys. These surveys are short and will not take much of your time at all. Your surveys at the end of each week (starting on your 7th day in the study) will be a little bit longer, so we wanted to give you a heads-up about that.

 Even though you will receive survey links through both email and text, you should only complete one survey per day. So, do not complete the survey from your email AND the survey from your texts on the same day -- just complete one of them, because they are the same. We are sending the link through both email and text just to make sure you see it each day. 

 After your first 4 weeks in the study, you will only be asked to complete 1 survey each week for the remaining 2 months. 

 Do you have any questions about the study?

Please take a moment, if you can, to add Jena Slaski (xxxxxxxx) to your email contacts list to make sure you receive your daily survey links, so they don't get caught in your spam or junk mail folder.

 ***Click "next" below to log the patient's responses***

End of Block: Post-Survey Script
