# Supplementary material for: Preventing opioid prescribing for low back pain using multimodal mechanical stimulation vs. TENS: a randomized-controlled trial
Source: Front Pain Res (Lausanne). 2025 Jul 10;6:1612572. doi: 10.3389/fpain.2025.1612572 (PMC12287057; doi:10.3389/fpain.2025.1612572)

**Supplement 4: Missing Data Handling Decisions Procedures**

**Missing Data Plan:**

Analysis will be using intention-to-treat for eligible enrolled patients, maintaining the same dataset of sociodemographic and low back pain (LBP) specific registration data for each area of outcome interest (opioid prescribing and use, and pain intensity and interference (function)). Subjects who did not record any baseline or outcome data will be removed from the relevant analysis. For example, any subjects who do not record any daily diaries cannot be analyzed in the opioid prescribing or use analysis or change in pain over time but will be included in the analysis of initial pain outcomes. Any subject who does not record an initial or follow-up Numeric Rating Scale Pain Intensity score outcomes will be excluded from acute pain analysis, but maintained in the opioid or pain interference change over time analysis.

Diary entries and item-level data will be analyzed for missing data to assess the need for imputation. When needed, NRS will be imputed with Last Carried Forward, and Pain Interference will be evaluated with a Linear Mixed Model. For loss to followup and random missing data, this mixed-effects model repeated measures (MMRM) approach will be used to provide implicit imputations of missing data for continuous outcomes.

Dose, Opioid Formulation, Source Evaluation (DOSE) tool:

“New prescription” outcomes use a response to the “source” prompt of “prescribed to me for this event”. If an opioid is endorsed with the same dose and pill number on multiple days, but the source differs, any endorsement of “to me for this event” will still be considered a new prescription as a binary outcome, thus no imputation or missing data will be needed.

“Use” outcomes are any opioid endorsed with dose and brand. When multiple opioids are endorsed with source and pill number on multiple days, but not pill dose, the dose is assumed to be the most common reported by the participant if 3 or more other instances of the same are recorded.

**PAIN INTERFERENCE**

The primary pain outcome for chronic patients is clinically important change in functional pain interference using the PROMIS 8a short form over time, calculating T-scores using HealthMeasures scoring (Northwestern.edu). For low back pain, the average minimally important clinical difference using Pain Interference will be 5, on the higher side of the 3.5-5.5 range reported by Chen et al. . <https://www.ncbi.nlm.nih.gov/pmc/articles/PMC4854267/>

PROMIS scores of 10 are one standard deviation, with the numbers between 61 and 65 being most sensitive for clinical change. For Pain Intensity, a low back pain score of 60 is considered moderate, and 70 is severe. For pain interference, a score of 55 is considered mild pain, and a low back pain T-Score of 57.7 – 65.4 crosswalks to an Oswestry Disability Index (ODI) rating of “moderate”, a T-score of 65.7-71.5 to “severe”, and a score of 71.9 or more “crippled”.[1] LBP resolution for patients with chronic pain >1 year will be considered categorically as compared to TENS, defined as both a decrease of 5 AND a change in category (e.g. from 70 to 60 (severe to moderate) or 60 to 50, and a continuous variable. Exploratory outcomes of “transformational pain reduction” will be defined as reductions of 2 standard deviations (20) over the duration of followup, and a reduction in VAS of 2.5 to compare with other rehabilitative therapies. Transition of acute to chronic pain will be evaluated based on pain of 55 or higher at 3 months (cLBP definition) for those endorsing pain <3 months at entry.

***Initial registration database review 10 days after completion of enrollment*:**

Participants were verbally screened for inclusion and exclusion criteria from an investigator guide sheet prior to informed consent, randomization and enrollment on a tablet. Supplement 3: Data Collection. The purpose of the study and opioid use reporting were discussed during informed consent.

**RE-ENROLLMENT:** For 20 subjects, the Qualtrics system did not recognize the format of the phone number or email entered at registration, resulting in failure of Qualtrics to remind patients via one or both modalities to enter that day, week, or month’s information. To fix the diary notification issue, subjects were re-enrolled remotely by the study coordinator and a new diary ID was automatically generated. During subsequent re-enrollment by the study coordinator to activate the daily reminder notifications, data fields were filled in with nonsense data or bypassed as the goal was just to activate the notification system. The intervention allocation remained that of the device dispensed at enrollment.

**Procedure:** unless noted, the ID and data from the first ID associated with the registration minimum dataset data (“Registration ID”) will be used; diary data will be merged to the registration ID from the time the re-enrollment ID is active. If duplicate diary days are entered during transition, the earlier of the two will be used. Numbers 10 9106 2783 4184 test registrations. 8560 no monthly or weekly.

| Reg ID | New ID | Enrollment | Re-E | Days to |  |
| --- | --- | --- | --- | --- | --- |
| 8962 | 9906 | 6/25/2022 | 6/27/2022 | 2 |  |
| 1525 | 5015 | 6/25/2022 | 6/28/2022 | 3 |  |
| 5393 | 2485 | 6/25/2022 | 6/27/2022 | 2 |  |
| 8491 | 2315 | 6/25/2022 | 6/27/2022 | 2 |  |
| 4050 | 596 | 6/25/2022 | 6/27/2022 | 2 |  |
| 7241 | 1435 | 10/3/2022 | 10/3/2022 | 0 |  |
| 3500 | 8134 | 10/14/2022 | 10/20/2022 | 6 |  |
| 9824 | 2826 | 10/18/2022 | 10/21/2022 | 3 |  |
| 8818 | 8653 | 12/5/2023 | 12/7/2023 | 2 |  |
| 9645 | 5370 | 1/10/2023 | 1/10/2023 | 0 |  |
| 1015 | 5033 | 1/10/2023 | 1/23/2023 | 13 |  |
| 6951 | 1725 | 2/17/2023 | 2/17/2023 | 0 |  |
| 7238 | 8422 | 2/20/2023 | 2/22/2023 | 2 |  |
| 5623 | 2396 | 3/6/2023 | 3/9/2023 | 3 |  |
| 8762 | 4268 | 4/14/2023 | 4/19/2023 | 5 |  |
| 2995 | 2805 | 5/26/2023 | 6/1/2023 | 6 |  |
| 5960 | 9088 | 8/23/2023 | 8/23/2023 | 0 |  |
| 8473 | 6618 | 10/3/2023 | 10/4/2023 | 1 |  |
| 5279 | 4807 | 10/31/2022 | 10/31/2022 | 0 |  |
|  |  |  |  |  |  |
|  |  |  |  |  |  |

**Pooling Stratified Analysis**

**ACUTE V. CHRONIC:** As prognosis, pain, and etiology differ between acute and chronic pain, the [The NIH BACPAC consortium](https://heal.nih.gov/files/2021-02/BACPAC%20Definition%20of%20cLBP%20%28BACPAC_2014%20Task%20Force%20Crosswalk%29%20%281%29.pdf) V1 Aug 4, 2020 minimum dataset includes 6 different time frames of pain, defining acute low back pain as pain lasting less than three months. Chronic pain studies have found an increased risk of persistent LBP after one year, and increased pain associated with 3 or more years of cLPB.

The FDA requested 6 months of follow-up for chronic patients, and we decided to use 3 months for acute pain participants. Participants populated identical registration data on a tablet stratifying participants into a 3 month or 6-month follow-up data collection program. After informed consent and before the participant was given the study intervention, the data collection tool prompted the enroller to verify “How long has the patient experienced ongoing back pain? (If greater than 3 months, switch to the chronic survey.)”

The enroller was then instructed to transition to the Chronic enrollment if the participant endorsed low back pain longer than 3 months. One patient was re-enrolled in the Chronic follow-up duration at this time.

| 3361 (A) | 3683 C | 12/5/2023 | 12/5/2023 | 0 |  |
| --- | --- | --- | --- | --- | --- |
|  |  |  |  |  | (acute->chronic after enrollment question. ID 3683 used throughout) |

Of the 60 patients verbally endorsing and enrolled in the Acute follow-up duration strata, on a later demographic question of “How long has low back pain been an ongoing problem for you?), 16 endorsed ongoing pain of longer than 3 months.

**Procedure:** For all outcomes in which acuity is a clinical consideration for pain, the pain duration reported in the registration minimum dataset will be used to stratify duration. As pain duration is not a recognized prognostic indicator of new opioid prescribing, the acute and chronic strata will be pooled. For consideration of completion of 3 or 6-month follow-up, adherence to the initially assigned computer data collection duration will be used, as these participants were not identified in time to extend their monthly data collection.

**PRIOR OPIOID USE:**

Some participants denied opioids at screening but then endorsed using an opioid in the registration minimum dataset. As many lay people do not understand which medications fall under the category of opioid, e.g. knowing that ibuprofen is not an opioid while tramadol is, a comprehensive opioid data collection tool was used in the registration dataset and the daily opioid diaries, which may account for the denial of opioids during screening but endorsing later.

**Procedure:** For all outcomes involving prior opioid use, a response of “no short-acting opioids” and “no long-acting opioids” recorded in the registration minimum dataset will be considered “opioid-naïve”. Participants endorsing one or more specific opioids and not endorsing “no short-acting opioids” and “no long-acting opioids” will be analyzed as a “prior user”. Opioid outcomes for prescribing will be followed weekly for 3 months.

**MIXED IBUPROFEN/ASPIRIN FORMULATIONS:**

In order to capture all potential types of opioids a participant might have in the house in the DOSE tool (Supplement 2), US brands, international, current and no longer prescribed were included.

To capture opioids prescribed or dispensed in other countries, a final category was made for “Other Short Acting [ex: Dihydromorphine, Nicomorphine, Oxycodone with Aspirin or Ibuprofen, Oxymorphone] “

Of the 60 enrolled in the Acute Group who verbally denied initiating opioid use for this episode of pain, 10 participants immediately endorsed those medications but no other opioids, and recorded “no short-acting opioids” and “no long-acting opioids”. On the data prompt in the enrollment tablet the words “Aspirin and Ibuprofen” were noted to start a new line:

Other Short Acting [ex: Dihydromorphine, Nicomorphine, Oxycodone with

Aspirin or Ibuprofen, Oxymorphone]

On daily opioid diaries, 30 people in the first 10 days endorsed this selection, putting in 325, 200, 400, or 600 and a number of pills correlating with 200mg/pill, dosing common for Aspirin and ibuprofen.

Oxycodone with ibuprofen or Aspirin are much less common in circulation than the frequency reported using this choice endorsement. To verify local trends, we consulted with a clinical opioid researcher at John’s Hopkins in the catchment area for the study. She denied any instance of a patient using any of the drugs in the "other short acting" option in her patient population or in the pre-op opioid weaning clinic. The study clinician contacted two subjects, who endorsed their intent to record ibuprofen.

**Procedure:** The category of “Other Short Acting [ex: Dihydromorphine, Nicomorphine, Oxycodone with Aspirin or Ibuprofen, Oxymorphone]” will be removed and the answers will not be considered in the opioid use, as the subjects were presumed to be responding to the Aspirin or Ibuprofen prompt.

***Blinded database review after completion of follow-up:***

**Exclusion/Inclusion Criteria:**

**Pain Severity:** Participants verbally endorsed the inclusion criteria of moderate to severe pain of (>=4 out of 10), the accepted level established by the International association of the Study of Pain and recently reendorsed and defended by the IMPAACT pain research guidelines.[Langford] After enrollment, participant pain recorded in the registration minimum dataset did not meet the NRS cut off inclusion criteria of the “pain now” or “pain past 24 hours” for 14 of 159 participants.

We consulted the literature to determine the proper approach when ineligible patients were mistakenly included. To ensure “**the decision to remove such patients is unbiased and not influenced by events that occurred after randomisation (and may therefore be affected by whether patients received experimental or control treatment), an independent adjudication committee blinded to treatment and outcome must systematically review each patient.**”[2]

We created an Independent Adjudication Committee by contacting colleagues on NIH PURPOSE pain listserv and prior NIDA scientific review group panels with experience in opioid research.

-A senior researcher in pediatric pain recommended including all who verbally endorsed moderate to severe pain.

-A researcher in opioid weaning noted that six participants did not reach the entry NRS of 4, but qualified as high impact chronic pain because “In the past three months, how often did you have pain?” was answered as most or every day, and Pain Interference >55s, indicating limited daily life or work activities on most days or every day.

-A Veteran’s Administration NIH pain investigator recommended excluding those who didn’t have moderate to severe pain on any metric, but also would have included four chronic pain patients who had pain for over two weeks, Pain Interference T scores >50, and endorsed the worst pain as a 3/5 or higher in the past 7 days.

-A PURPOSE pain researcher recommended completing the analysis including and excluding those who did not record NRS levels of 4 or more and reporting results of one in supplemental material.

In the most recent Back Pain Research Task Force,[3] Deyo et al recommend using the sum of the 7 day average Pain Intensity, PROMIS Physical Function and Pain Interference scores from the low back pain minimum dataset to define LBP impact ranging from 8 - 28-34=moderate, >=35 severe. They note that the cutoffs were determined in a “rather severely affected” sample of 218 patients presenting for epidural administration, and recommend “simply reporting actual scores, along with any categorization that investigators may choose.

**Procedure:** We calculated intention to treat (n=159) including all who had verbally acknowledged moderate to severe pain >=4 out of 10. For pain outcomes, we will analyze a subset of more severely affected participants with a Back Pain Research Task Force RTF >27 (n=110).

**BMI:** Exclusion criteria was set in the pilot study at “BMI>=30 or device won’t fit” based on the 45” prototype neoprene belt maximal would hold the device against the back with pressure without causing discomfort. The final 54” Neoprene belt is indicated up to BMI<50. Fit was used by clinic staff. One subject with a BMI of 60 was randomized to DuoTherm but was unable to attach the device as directed based on abdominal girth and diary use data (exclusively reported lying on the device in bed).

**Procedure:** Participant 6468 will be excluded from analysis. Additional analyses controlling for BMI will be conducted. Future studies are advised to include a calculator embedded in the programming prior to distributing a device.

**Repeat Diary Data**

For weekly or monthly surveys, if duplicate entries within hours or days are recorded the earlier of the surveys will be used if substantially equivalent (e.g. they forgot if they had filled it out and re-entered). If one survey was substantially more complete as measured by the “PERCENT” column indicating the percent of the survey which was completed, the survey with more complete data was used.

For daily surveys, if the day prior was missed and an entry is made on the following day before noon, it will be used as the previous day.

If two entries were for the same day at similar times, the earlier of the surveys will be used if substantially equivalent. If one was partially filled out, the survey with more complete data was used UNLESS the second survey added opioid information, in which case opioid data was added to prior more complete information. If the second included opioids and was just as complete, it was used.

For Example:

First diary at: 22:30 - no opioids, pain diary complete

Second entry at : 23:15 oxycodone, no pain diary

**Procedure:** Data used: Second oxycodone, First pain diary information

**Duplicate Data:** No entries were combined; 73 of 4570 were culled due to redundant triggering of the reporting system or a redundant entry.

**Duration of Opioid Use**

**Missing Data:**

**DOSE tool**: The skip-logic algorithm for the DOSE tool took participants to the next choice (dose, number, source) with a forward button or tab. When a prompt was skipped to go to the next question, missing data was assigned #NULL in the registration dataset, and -99 in diary datasets. When the back button was pressed, the endorsement of the opioid was not erased, and a #NULL was not recorded for pill dose, number of pills or source. Confirmation of an accidental endorsement could be confirmed if participants then endorsed “no opioid use”.

**Procedure:** When an opioid was endorsed a single time for a respondent without a response or missing data flag for pill dose, number, or source, AND when there were no other opioids for the participant, with 10 or more diary days recorded, the single entry was presumed to be in error and no MME were attributed.

4570 diary days recording any opioid use were recorded prior to time stamp consolidation. This happened for two entries, and the daily diaries were all filled out as “no opioid use”. (n=2, 125, 4003).

**Mg Morphine Equivalents**

The doses were calculated using the HHS morphine equivalents page. Initially fentanyl patch dosing was included at 25 ug/hr fentanyl patch X 24 hrs = 600 ug/day fentanyl = 60 mg/day oral morphine milligram equivalent. Subject matter experts on opioid use and opioid surveys noted that patches were rarely if ever prescribed for outpatient LBP exacerbations; the FDA medical device development toolkit validation review group also felt fentanyl and oral medications treating opioid use disorder were not relevant for an acute pain intervention study.

**Procedure:** MME will be calculated without Fentanyl patch use or medication assisted treatment (buprenorphine).

**Missing Data, DOSE**:

When a weekly survey denoted multiple strengths and pills taken, the average was recorded. For example, Codeine has 4.5MME for a 30mg tab. An entry from a weekly diary saying

| Codeine | 300/30,300/60 | 6 |  |
| --- | --- | --- | --- |
|  |  |  |  |

Would be averaged as 3 30mg tablets (13.5MME) and 3 60mg (27MME), for a total 50.5MME.

The response “9 or more” was calculated as 9.

When an opioid was endorsed but pill dose or pill number had #NULL or -99, the mode response for that participant was added if there were at least 3 other opioid days recorded N=4, 9120,3683,4382,7086 ).

When an opioid was endorsed with dose and pill number on multiple days, but source differed, any use of the source “to me for this event” was considered a new prescription. (n=1, 7181 had 2 entries saying “to me for this event” and 30+ endorsing “to me for another event” but was considered new prescription.

When an opioid has both a long and a short acting form, and the participant entered the same name, dose, pill number and source under both SA and LA, the assumption was that the participant only took one set and was unsure where to enter the data. MME was only recorded once. (n=2, 6933, 9120)

Reference Document 1. Inclusion/Exclusion Training Guide:


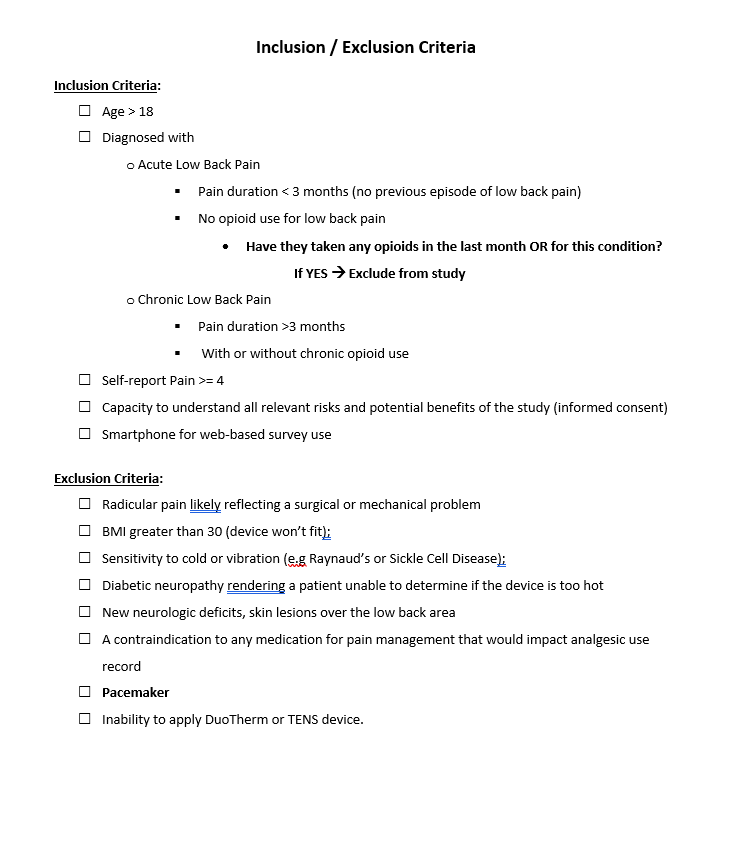


Reference Document 2:
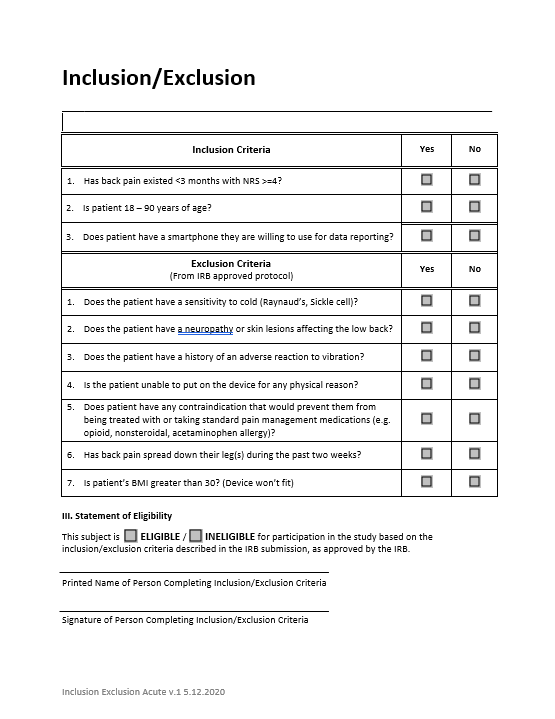

Supplement: Supplementary file 4 [file Datasheet4.docx]
