# Supplementary material for: Preventing opioid prescribing for low back pain using multimodal mechanical stimulation vs. TENS: a randomized-controlled trial
Source: Front Pain Res (Lausanne). 2025 Jul 10;6:1612572. doi: 10.3389/fpain.2025.1612572 (PMC12287057; doi:10.3389/fpain.2025.1612572)
Supplement: Supplementary file 5 [file Datasheet5.docx]

**Supplement 5: Data Sharing Statement**

**Plan to Share Individual Participant Data (IPD)?**

Yes

**IPD Plan Description**

Individual participant data that underlie the clinical results reported in any publications, after deidentification, will be provided to the HEAL NIH dataset for low back pain (BACPAC) within 12 months of publication of the results. Data will be available 36 months after publication to achieve approved aims of any researcher who provides a methodologically sound proposal. Proposals should be directed to info@mmjlabs.com. To gain access, data requestors will need to sign a data access agreement.

**IPD Sharing Access Criteria**

Automatic to HEAL Data Sharing Registry. Researchers providing approved methodologically sound proposals

**IPD Sharing Time Frame**

36 months after publication of results

**IPD Sharing Supporting Information Type**

Study Protocol – included in public eSupplement

Statistical Analysis Plan (SAP) – included in publication

Clinical databases in .csv files
