# Supplementary material for: Preventing opioid prescribing for low back pain using multimodal mechanical stimulation vs. TENS: a randomized-controlled trial
Source: Front Pain Res (Lausanne). 2025 Jul 10;6:1612572. doi: 10.3389/fpain.2025.1612572 (PMC12287057; doi:10.3389/fpain.2025.1612572)
Supplement: Supplementary file 6 [file Datasheet6.docx]

**Supplement 6: Development of Predictive Neural Network for Disability Resolution**

The goal of the the study was not just to compare two different pain devices, but to understand the novel technology of the multimodal M-stim device and identify the optimal candidates for pain reduction and potential reversal of chronic pain. As an initial pivotal study of a novel device with a heterogeneous population, …

**Neural networks** are highly complex interactions of adjacent nerves and connections with predictable patterns of behavior in response to stimuli. In computer science, neural networks construct layers of “neurons” that create interactions between provided data. The parameters of the model are optimized through training of forward and backward vectors of relationships (multivariable calculus) to reach a model with the lowest error when tested against the actual data. Neural networks can predict which subject characteristics are most associated with outcomes of interest. To predict Week 13 PROMIS T-scores, a neural network was chosen because interactions between neuron features provide the possibility to discover more complex patterns within the data.

Networks vary in number of hidden layers and neurons. We had one input layer (the dataset) converted to vector form; we used 2 hidden layers with 64 neurons in the first and 32 in the second. The activation function used was rectified linear unit and the objective loss function was mean squared error (MSE). To help reduce errors, an ensemble of 5 feed-forward neural networks were created with two hidden layers. The predictions for a given PROMIS T-score were produced through the average of the models. Dropout and early stopping were used to reduce overfitting. Optimal train/test split was found to be 90/10. The model produces Week 13 PROMIS T-score predictions with a Mean Average Error of 3.82. This proves the model has significance as guessing the mean values for y_test gives a MAE of approx. 6.91. The r-squared graph is as shown:


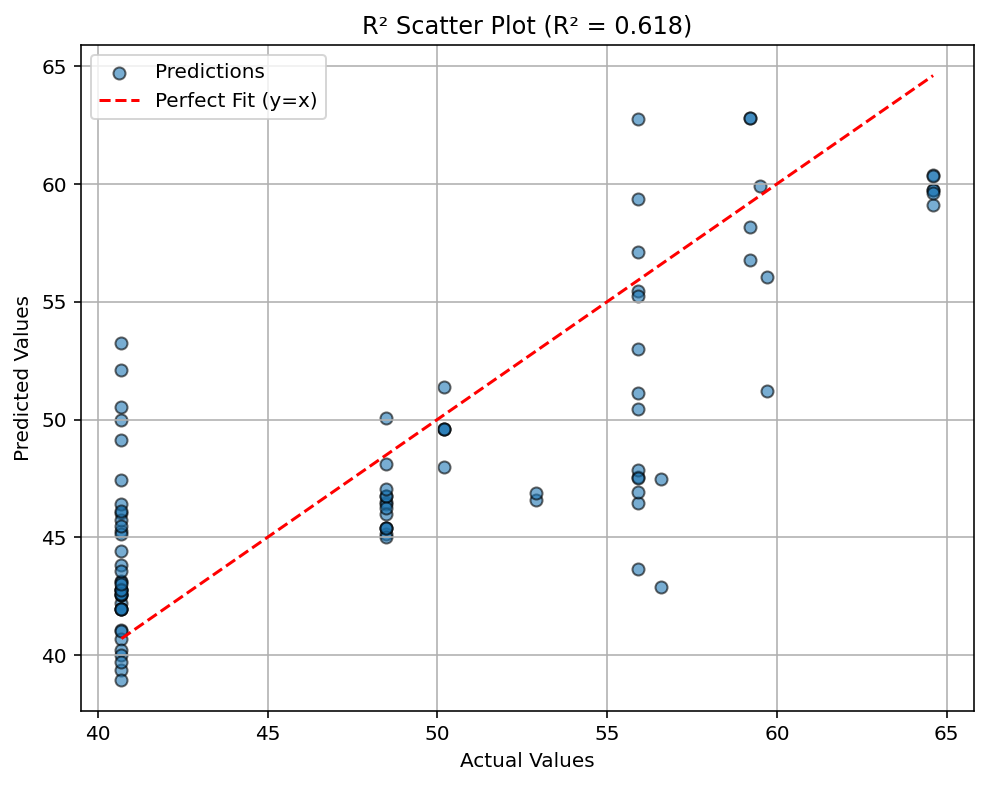
It’s clear that true values of PROMIS scores lead to a very clustered domain. The main limitation of the model is this underlying distribution of the data.

With a model that predicts PROMIS T-Scores on data of patients with significant outcomes, the feature importance can be extracted to determine what attributes most effect the model’s decision. The SHAP values are displayed below:


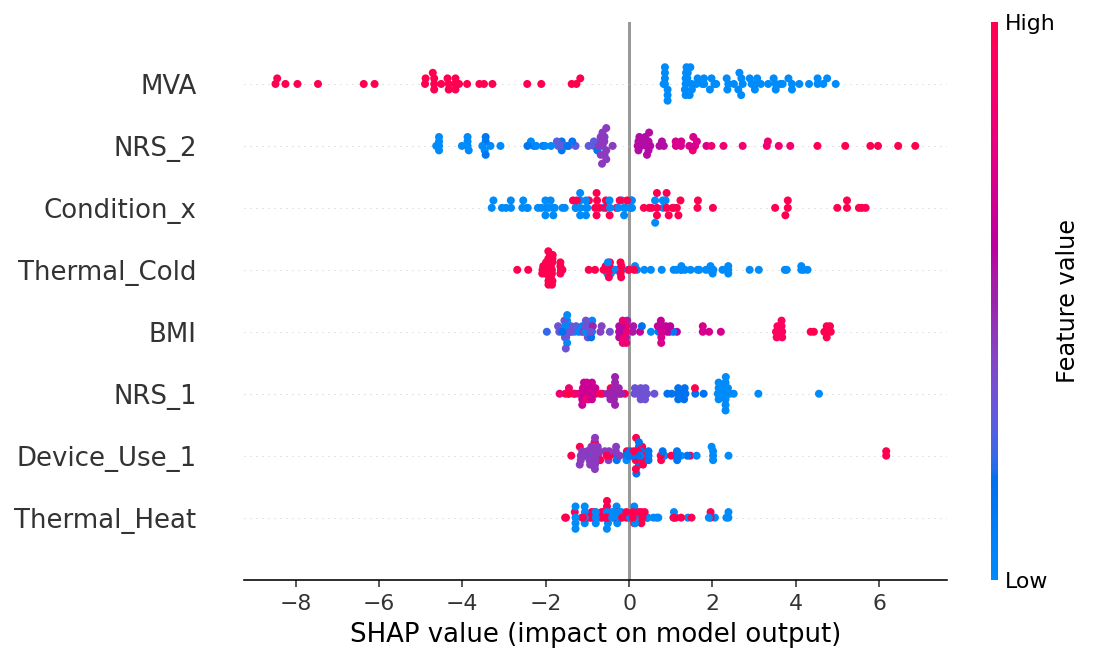


Predictions of week 13 PROMIS score for both conditions are shown, with each dot representing an individual subject.

Colors show whether the feature made a large or small impact on week 13 PROMIS scores, where negative values equate to lower ending PROMIS scores (better outcomes). For example, MVA red (1) had an MVC, and these subjects were more likely to have a better final score. blue=0 (no MVC) was highly predictive of better improvement in week 13 scores. MVA Red = Yes Blue = NO; NRS_2 = 24 hour pain intensity, continuous variable. Condition 1=blue, condition 2=red. Thermal use of cold: Red = yes used cold Blue = No use of cold; BMI continuous variable: Red high, blue low. NRS_1 Pain improvement >=3 continuous red higher. Device use

To predict what contributes to significant change in disability, only those with chronic pain (Pain_Length >= 4, meaning 6 or more months, >1 year, or >5 years) were included. the change in “pain now” was included at a higher level of pain intensity over the 13 weeks than the 10 day model. Thus, only **(ΔNRS_1 =<-3) OR NRS_2=<-2 or -3)** improvement were included in the model.

There are some immediate trends observable from how the model makes its prediction. Clearly, MVC (Motor Vehicle Collision), a binary variable, has a significant impact on predicted outcomes. Interestingly, a value of 0 (no motor accident) leads to higher predicted PROMIS T-scores. This same trend can be observed from Thermal_Cold, another binary variable. A value of 0 (no cold applied) seems to be associated with higher PROMIS T-scores. Device_Use_1 is a scalar value of time in minutes that a device was used. The model suggests that more time used decreases PROMIS T-scores. Furthermore, lower BMI seems to be associated with lower predicted PROMIS T-scores.

Some attributes seem to have mixed influence on the model, such as Thermal_heat. The clustered values support this, as well as the SHAP data that puts it at the bottom of feature importance.
